# The Role of Endogenous Neuroprotective Mechanisms in the Prevention of Retinal Ganglion Cells Degeneration

**DOI:** 10.3389/fnins.2018.00834

**Published:** 2018-11-15

**Authors:** Marita Pietrucha-Dutczak, Marialaura Amadio, Stefano Govoni, Joanna Lewin-Kowalik, Adrian Smedowski

**Affiliations:** ^1^Chair and Department of Physiology, School of Medicine in Katowice, Medical University of Silesia, Katowice, Poland; ^2^Department of Drug Sciences, Section of Pharmacology, University of Pavia, Pavia, Italy

**Keywords:** retinal ganglion cells, optic neuropathy, endogenous neuroprotection, cell survival, stress-response

## Abstract

Retinal neurons are not able to undergo spontaneous regeneration in response to damage. A variety of stressors, i.e., UV radiation, high temperature, ischemia, allergens, and others, induce reactive oxygen species production, resulting in consecutive alteration of stress-response gene expression and finally can lead to cell apoptosis. Neurons have developed their own endogenous cellular protective systems. Some of them are preventing cell death and others are allowing functional recovery after injury. The high efficiency of these mechanisms is crucial for cell survival. In this review we focus on the contribution of the most recently studied endogenous neuroprotective factors involved in retinal ganglion cell (RGC) survival, among which, neurotrophic factors and their signaling pathways, processes regulating the redox status, and different pathways regulating cell death are the most important. Additionally, we summarize currently ongoing clinical trials for therapies for RGC degeneration and optic neuropathies, including glaucoma. Knowledge of the endogenous cellular protective mechanisms may help in the development of effective therapies and potential novel therapeutic targets in order to achieve progress in the treatment of retinal and optic nerve diseases.

## Introduction

**FIGURE 1:**
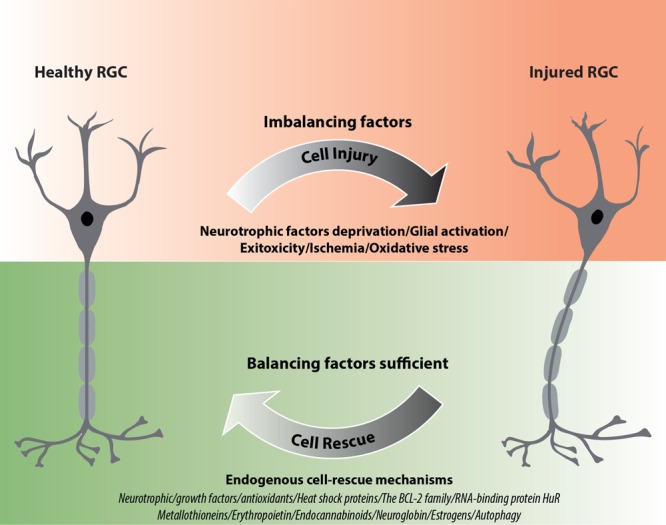
Schematic diagram illustrating the impact of endogenous neuroprotective mechanisms on RGC rescue after injury. ^∗^If factors representing the endogenous rescue-mechanisms are sufficiently activated they can allow functional recovery of RGCs; however, if these mechanisms are not efficient enough, irreversible cell death may occur.

Retinal neurons are considered to be a part of the central nervous system (CNS). As a consequence, they share several properties with CNS neurons, including the inability to undergo spontaneous regeneration in response to damage. Since there is no possibility to replace cells that become non-functional due to damage or aging, these long-living cells are likely endowed with specific mechanisms to protect them from both intracellular and environmental stress (Hayashi and Takagi, 2015). Retinal neurons, including retinal ganglion cells (RGCs), are exposed to similar stressors as observed in other cell types, i.e., UV radiation, high temperature, ischemia, hypo- or hyperoxic conditions, harmful microbes, allergens and environmental pollutants, all factors able to induce reactive oxygen species (ROS) production and/or release (Bouayed and Bohn, 2010). In addition, retinal neurons are exposed to the glutamate, which in excess concentration is a neuron-specific toxin. Subsequently, ROS production results in genes expression alteration, protein misfolding and other events leading to cell stress with apoptosis as the terminal process. Since the technologies allowing CNS repair by cell replacement are still under development, the efficiency of the endogenous cellular protective systems is a key factor for neuronal apoptotic susceptibility and survival (Bakalash et al., 2002; Wei Y. et al., 2011). As a consequence of this, the neurodegeneration can be described as an imbalance between protective and damaging factors affecting RGCs (Figure 1).

Retinal ganglion cells projections form the optic nerve (ON) and conduct the visual signal from the retina to visual centers in the brain (i.e., lateral geniculate nuclei). RGCs are the site of numerous primary retinal and optic nerve pathologies among which glaucoma, ischemic optic neuropathy and Leber’s hereditary optic neuropathy (LHON) are the most common. Moreover, there are pathologies with ocular signs, such as diabetes (Gerber et al., 2015; Wan et al., 2017), Alzheimer’s disease (AD) (Hart et al., 2016) and brain tumors (Tieger et al., 2017). Damage to RGCs or the ON results in impairment of visual signal propagation and subsequent progressive visual field defects. Since the retina represents a highly organized structure, with neighboring cells closely interacting with each other, death of a single RGC can induce self-perpetuating processes affecting survival of surrounding cells; these detrimental events are related mostly to the release of intracellular glutamate from the dying cells that triggers an excitotoxic cascade (Chang and Goldberg, 2012). Therefore, impairment of RGC endogenous neuroprotective mechanisms and increased RGC apoptosis can participate in spreading of pro-death signals resulting in progressive damage to the retinal cells.

Endogenous neuroprotective mechanisms in neurons, including RGCs, can be classified in various categories. Neurotrophic factors and both enzymatic and non-enzymatic mechanisms of ROS scavenging are well-known players in RGC homeostasis. In addition, systems of misfolded protein degradation (i.e., autophagy) and mechanisms controlling gene expression are gaining increasing attention and represent potentially interesting targets for therapy and/or prevention of neurodegeneration. Here, we summarize and describe the most recently studied endogenous neuroprotective factors involved in RGC survival, which include (a) neurotrophic/growth factors (neuronal, vascular, or both), (b) processes regulating the redox status, and (c) other factors, including pathways regulating apoptosis/cell death and neuroinflammation modulators (Figure 1).

## Neurotrophic/Growth Factors (Neuronal, Vascular or Both)

### Neurotrophic Factors

Neurotrophic factors regulate the development and survival of neurons. They seem to be involved in the endogenous neuroprotection of RGCs. A variety of studies have reported that neurotrophic factors, particularly nerve growth factor (NGF), brain-derived neurotrophic factor (BDNF), neurotrophin-3 (NT-3), neurotrophin-4/5 (NT-4/5), glial cell-derived neurotrophic factor (GDNF), and ciliary neurotrophic factor (CNTF), protect RGCs in various models of ON injuries (Johnson et al., 2011; Domenici et al., 2014; Gupta et al., 2014; Table 1). In agreement with their well-known role in maintaining neuronal homeostasis, neurotrophic factors have been proposed as novel therapies for various neurodegenerative diseases, however, outcomes of known clinical trials were not satisfactory, presenting only partial or no expected effects (Greenberg et al., 2009; Allen et al., 2013; Shruthi et al., 2016). Interestingly, some CNS pathologies, such as AD, Parkinson disease and Huntington disease, display ocular signs and alterations within RGCs (Batcha et al., 2012; Ramirez et al., 2017); thus, for these neurodegenerative diseases, neurotrophic factors promoting the survival of neurons may be of beneficial at both the central and ocular levels.

**Table 1:** Expression of neurotrophins and their receptors in different RGC degeneration models and human glaucoma (↑ upregulation; ↓ downregulation).

Factor	Model	Observation	Methods Used to Analyze	Reference
**BDNF**	**MOUSE**			

	Microbeads injection	ONH: 5 months – nsa 1 year – ↓ BDNF (WT < BDNF^-/+^)	Western blotting, ELISA	Gupta et al., 2014, 2016
	NMDA-induced retinal degeneration	14 and 21 days – ↓ BDNF	IHC	Jindal et al., 2017
	Model of spontaneous glaucoma (DBA/2J)	With age – ↓ BDNF, ↓ TrkB	Western blotting	Chou et al., 2018

	**RAT**			

	Episcleral cauterization	Retina: 28 days – ↑ BDNF, ↑ mRNA BDNF; TrkB – nsa; ↑ p75	Western blotting, IHC, RT-PCR	Rudzinski et al., 2004
	Retinal ischemia (acute IOP elevation)	RGC: 4 h – ↑ BDNF RGC and ONH: 4 h – ↑ Trk B	IHC	Pease et al., 2009
	Hypertonic saline episcleral injections into aqueous veins	ONH: 7 days – ↓ BDNF 14 days – ↑ BDNF (glia) Retina: 7 and 14 days – ↓ BDNF >14 days – ↑ BDNF (RGC soma)	IHC	Johnson et al., 2000
	Carotid artery occlusion	Retina: 3 and 14 days – ↓ BDNF mRNA 3 days – ↑ BDNF 14 and 60 days – ↓ BDNF (GCL) 3, 14, and 60 days – ↓TrkB (GCL)	Western blotting, IHC, ELISA	Guo et al., 2014

	**HUMAN**			

	Tissues from fresh post-mortem glaucoma subjects	ONH: ↓ BDNF	Western blotting, ELISA	Gupta et al., 2014, 2016

**NGF**	**RAT**			

	Episcleral cauterization	Retina: 7 days – ↑ NGF (transient); ↑ Trk A	Western blotting, IHC, RT-PCR	Rudzinski et al., 2004
	Hypertonic saline injection	35 days – ↑NGF; ↑ NGF mRNA; ↑TrkA; ↑p75	ELISA, RT-PCR, IHC	Coassin et al., 2008
	Carotid artery occlusion	Retina: 3 and 14 days – ↓NGF mRNA 3 and 14 days – ↓TrkA	Western blotting, IHC, ELISA	Guo et al., 2014

	**HUMAN**			

	POAG	Serum: early and moderate glaucoma – ↓ NGF advanced glaucoma – nsa	ELISA	Oddone et al., 2017

**CNTF**	**MOUSE**			

	NMDA-induced retinal degeneration	14 and 21 days – ↑CNTF	IHC	Jindal et al., 2017

	**RAT**			

	Carotid artery occlusion	Retina: 3 and 14 days – ↑CNTF mRNA	Western blotting, IHC, ELISA	Guo et al., 2014
	Laser photocoagulation	14 days – ↑CNTF	IHC, Immunoblot	Ji et al., 2004

	**HUMAN**			

	POAG	Lacrimal fluid – ↓CNTF Aqueous humor – ↓CNTF	ELISA	Shpak et al., 2017

**GDNF**	**MOUSE**			

	NMDA-induced retinal degeneration	14 and 21 days – ↑GDNF	IHC	Jindal et al., 2017

	**RAT**			

	Carotid artery occlusion	Retina: 3 and 14 days – ↓GDNF mRNA	Western blotting, IHC, ELISA	Guo et al., 2014

**NT4/5**	**RAT**			

	Hypertonic saline episcleral injections into aqueous veins	ONH: 7 days – ↓ NT4/5 14 days – ↑ NT4/5 (glia) Retina: 7 and 14 days – ↓ NT4/5 >14 days – ↑ NT4/5 (RGC soma)	IHC	Johnson et al., 2000

**NT-3**	**RAT**			

	Episcleral cauterization	Retina: 28 days – nsa; ↑ Trk C (Müller cells but not in RGC)	Western blotting, IHC, PCR	Rudzinski et al., 2004
	Carotid artery occlusion	3 and 14 days – ↓ NT-3 mRNA 3 and 14 days – ↓ TrkC	Western blotting, IHC, ELISA	Guo et al., 2014

*nsa, no significant alterations; RGC, retinal ganglion cells; GCL, ganglion cell layer; ONH, optic nerve head; POAG, primary open-angle glaucoma; BDNF, brain-derived neurotrophic factor; NGF, nerve growth factor; CNTF, ciliary neurotrophic factor; GDNF, glial cell line-derived neurotrophic factor; NT-4/5, neurotrophin-4/5; NT-3, neurotrophin-3; Trk A/B/C, the tropomyosin kinase receptor A/B/C; IHC, immunohistochemistry; RT-PCR, reverse transcription*-*polymerase chain reaction.*

**FIGURE 2:**
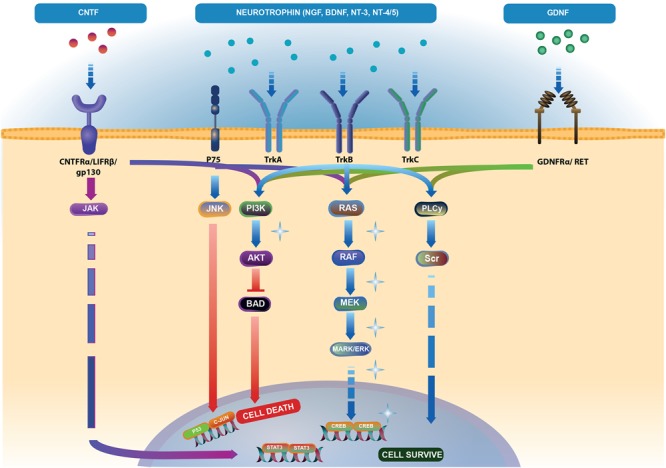
Neurotrophic factor (NTFs) signaling pathways. Brain-derived neurotrophic factor (BDNF), nerve growth factor (NGF), neurotrophin-3 (NT-3), and neurotrophin-4/5 (NT-4/5) bind to two families of receptors. Tropomyosin kinase (Trk) receptor binds with high affinity to promote cell survival via phospholipase C-γ (PLC-γ), phosphoinositide-3 kinase (PI3K) and mitogen-activated protein kinase (MAPK) pathways (light blue arrows). Binding of NTFs to low affinity p75 receptor activates cell death through the JNK pathway (light blue and red arrows). Ciliary neurotrophic factor (CNTF) binding to CNTFRα receptor and two subunits – gp130 and leukemia inhibitory factor (LIFRβ) activate the Janus kinase/signal transducer and activator of transcription (JAK/STAT) (blue-violet arrows), MAPK and PI3K pathways (dark blue arrows). Binding of glial cell line-derived neurotrophic factor (GDNF) to the GDNFα receptor and tyrosine kinase RET receptor stimulates PLC-γ, MAPK and PI3K pathways (green arrows). Akt controls the activities of several proteins important in promoting cell survival, including substrates that directly regulate the caspase cascade, such as BAD. Phosphorylated BAD prevents its proapoptotic actions (Skaper, 2008) (red arrows). The pathway illustration was based on Reactome (https://reactome.org/ PMID: 29145629, PMID: 29077811). Represents the pathways responsible for endogenous cell-rescue mechanisms in glaucoma; these pathways were significantly activated when IOP was elevated, but decreased to baseline levels when IOP was lowered (Levkovitch-Verbin et al., 2007; Levkovitch-Verbin, 2015).

Neurotrophins can bind to different receptors and transduce diverse intracellular signals, finally leading to opposite outcomes – death or survival. For instance, the binding of a neurotrophin to the tyrosine-receptor kinase (Trk) promotes cell survival, whereas interaction with p75 NT receptors (p75NTR) induces apoptosis (Kimura et al., 2016; Figure 2).

#### Brain-Derived Neurotrophic Factor (BDNF)

Neurotrophin deprivation, mainly BDNF, is considered as one of the leading causes of RGC death in glaucoma. BDNF is synthesized in the superior colliculus and the lateral geniculate nucleus and retrogradely transported to the RGC bodies. Moreover, this neurotrophic factor is produced locally in RGCs (Herzog and von Bartheld, 1998). Exposure to high IOP induces a two-phase response (a decrease followed by an increase) in BDNF and NT4/5 expression in RGCs and in the ON head glia, suggesting the participation of these neurotrophins in endogenous neuroprotective responses of RGCs (Vecino et al., 1999; Johnson et al., 2000; Table 1). It is widely accepted that BDNF upregulation is an early response in RGCs that undergo axonal injury (Kimura et al., 2016). Several studies indicated that the therapeutic effects of different neuroprotective agents in promoting RGC survival are related to their induction of retinal BDNF expression (Bai et al., 2010; Pietrucha-Dutczak et al., 2017; Chou et al., 2018). Our previous studies also confirm these results. Application of extracts from pre-degenerated peripheral nerves stimulate RGC survival through induction of endogenous retinal BDNF expression in glaucoma (Pietrucha-Dutczak et al., 2017). It is important to note that BDNF levels in the serum and tears of glaucoma patients are significantly lower than in control subjects, suggesting that deficits in this neurotrophin may participate in RGC death in glaucoma (Ghaffariyeh et al., 2009, 2011; Oddone et al., 2017). Furthermore, BDNF inhibits the osmotic swelling of Müller cells and bipolar cells (Berk et al., 2015) and upregulates the glutamate/aspartate transporter (GLAST) and Glutamine Synthetase (GS) expression in Müller cells in the mouse retina, increasing glutamate uptake during hypoxia (Dai et al., 2012). To our knowledge, no clinical trials evaluating the potential therapeutic effects of BDNF in glaucoma have been conducted so far, however, there have been many attempts and future plans in developing BDNF-based therapies.

#### Nerve Growth Factor (NGF)

**FIGURE 3:**
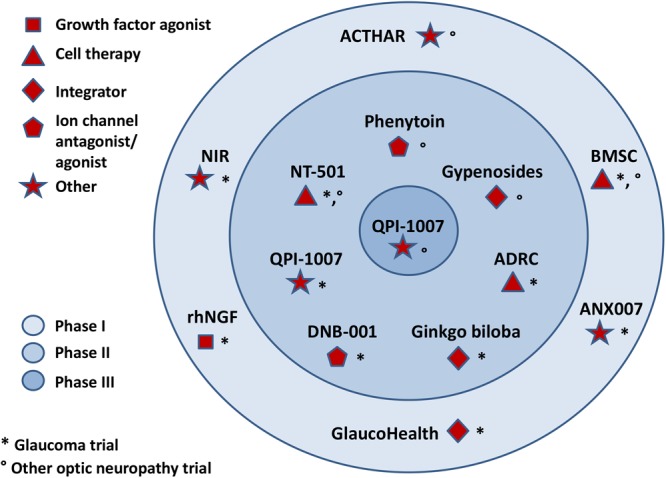
Major clinical trials for optic neuropathy therapeutics, including glaucoma.

Nerve growth factor is another important growth factor affecting the survival of nerve cells. NGF deprivation can lead to apoptosis preceded by impairment of glucose uptake, an increase in ROS production, activation of c-Jun N-terminal kinase (JNK) and its downstream target c-Jun, and caspase activation (Freeman et al., 2004; Lomb et al., 2009). Mature NGF (mNGF) is derived from its precursor form (proNGF) after conversion by a protease cascade within the extracellular space (Bruno and Cuello, 2006). It has been suggested that disturbance in proNGF to mNGF turnover can be involved in neurodegeneration, such as AD, as well as seizures, and cerebral ischemia (Fahnestock et al., 2001; Bruno and Cuello, 2006). NGF is produced and utilized by RGCs, bipolar cells and glial cells (Roberti et al., 2014) and can be involved in neuroplasticity of neurons in the visual cortex and geniculate nucleus (Maffei et al., 1992). Several studies indicate that NGF protects RGCs after ON ischemia or transection, ocular hypertension and glaucoma (Colafrancesco et al., 2011; Roberti et al., 2014; Aloe et al., 2015; Chen Q. et al., 2015). The downregulation of NGF and NGF-receptor expression in the retina and ON is reduced by ocular application of NGF in a rat model of glaucoma and protects animals from neurodegeneration (Colafrancesco et al., 2011). Moreover, NGF, similarly to BDNF, prevents the osmotic swelling of rat Müller glia, by a double action: directly, by activating autocrine/paracrine FGF signaling, and indirectly, by inhibiting the swelling of bipolar cells through induction of cytokine release from Müller cells. The inhibitory effect is mediated by activation of TrkA but not p75NTR (Garcia et al., 2014). The binding of NGF to TrkA in RGCs promotes their survival, while the binding of NGF to p75NTR is mainly responsible for pro-apoptotic responses (Figure 2); however, the binding of NGF to both TrkA and p75NTR at the same time leads to RGC survival, since p75NTR acts in concert with TrkA-helping receptor (Wang H. et al., 2014). In the context of potential application of NGF in glaucoma, the safety of an 8-week treatment with 180 μg/ml recombinant human NGF (rhNGF) eye drop solution was tested in a masked, randomized, vehicle-controlled, phase Ib trial of 60 participants with chronic POAG (NCT02855450); for now, the conclusions of this study have not yet been published (Table 2 and Figure 3).

**Table 2:** Glaucoma neuroprotective drug development pipeline.

Agent	Administration	Agent mechanism class	Mechanism of action	ClinicalTrials.gov identifier	Phase/Status	Sponsor	Start date
rhNGF	Eye drops	Neurotrophic factors	Pro-survival/ neuroprotection	NCT02855450	Ib/Recruiting	Dompé Farmaceutici S.p.A.	December 2016
NT-501 (CNTF-secreting cells)	Implant	Cell therapy/ neurotrophic factors	Pro-survival/ neuroprotection	NCT02862938	II/Recruiting	Stanford University	June 2016
Ginkgo biloba extract	Oral	Antioxidant/ neuroprotection	Antioxidant/ improving ocular blood flow	NCT02376114	II	Maisonneuve-Rosemont Hospital	August 2011
GlaucoHealth^1^	Oral	Antioxidant/ neuroprotection	Antioxidant/Anti-inflammatory	NCT02984813	I/Active, not recruiting	The New York Eye and Ear Infirmary	April 2016
BMSC	Intraocular injection	Stem cells	Replacement of damaged cells	NCT03011541	Not applicable/ Recruiting	MD Stem Cells	January 2016
BMSC	Intraocular injection	Stem cells	Replacement of damaged cells	NCT01920867	Not applicable/ recruiting	MD Stem Cells	August 2013
BMSC	Intraocular injection	Stem cells	Replacement of damaged cells	NCT02330978	I/Recruiting	University of Sao Paulo	January 2014
ADRC	Intraocular injection	Stem cells	Replacement of damaged cells	NCT02144103	II/Recruiting	Kremlin Hospital^2^	May 2014
ANX007	Intraocular injection	Other (biological – antibody)	C1q inhibition	NCT03488550	I/Recruiting	Annexon, Inc.	March 2018

*There are 1,287 interventions/treatments registered in ClinicalTrials.gov for glaucoma (accessed May 31, 2018), of which 9 agents may exert direct neuroprotective effects on the retina and/or optic nerve. See the text for further details on the classes. ^*1*^Alpha lipoic acid, citicoline, Co-enzyme Q10, Ginkgo biloba extract, grape seed extract, N-acetyl-cysteine, curcumin, and green tea extract. ^*2*^Central Clinical Hospital w/Outpatient Health Centre of Business Administration for the President of Russian Federation. rhNGF, recombinant human NGF; BMSC, autologous bone marrow-derived stem cells; ADRC, adipose-derived regenerative cells.*

#### Glial Cell Line-Derived Neurotrophic Factor (GDNF)

Glial cell line-derived neurotrophic factor is secreted by glial cells and binds to the GDNF-α receptor and tyrosine kinase RET receptor (Figure 2). RET is expressed in the inner nuclear layer and ganglion cell layer of the mammalian retina, whereas GDNF-α is expressed in RGCs, Müller glia, and amacrine cells (Yan et al., 1999; Airaksinen and Saarma, 2002). GDNF promotes axonal regeneration in the CNS and prevents apoptosis through the phosphoinositide-3 kinase (PI3K), mitogen-activated protein kinase (MAPK) and Src kinase pathways (Airaksinen and Saarma, 2002; Figure 2). GDNF upregulates GLAST in Müller glia and may indirectly protect RGCs by enhancing glutamate uptake (Koeberle and Bähr, 2008). It has been suggested that GLAST impairment may be involved in the pathogenesis of glaucoma (Kimura et al., 2016). To our knowledge, no clinical trials evaluating the potential therapeutic effects of GDNF in glaucoma have been conducted so far.

#### Ciliary Neurotrophic Factor (CNTF)

Ciliary neurotrophic factor binds to CNTFRα receptors and two signal transducing transmembrane subunits – gp130 and leukemia inhibitory factor (LIFR). This complex activates Janus kinase/signal transducer and activator of transcription (JAK/STAT), mitogen-activated protein kinase (MAPK)/ERK, and PI3/Akt signaling pathways (Kimura et al., 2016; Figure 2). CNTF is expressed in the neural retina, retinal pigmented epithelium and ON head (Beltran et al., 2003; Liu et al., 2007). The neurotrophic properties of CNTF were tested in several animal models of retinal diseases (Pease et al., 2009; Rhee et al., 2013; Mathews et al., 2015) and are currently under evaluation in clinical trials (Tao, 2006; Kauper et al., 2012; Birch et al., 2013, 2016; Chew et al., 2015). It has been suggested that the neuroprotective effects of this factor are mediated especially by Müller cells that directly respond to CNTF by releasing other neurotrophic factors such as bFGF (Wen et al., 2008). Furthermore, CNTF increases secretion of NT3 and decreases VEGF, IL8, and TGFb2 levels in primary cultures of human fetal retinal pigmented epithelial cells (Li et al., 2011). After glaucoma induction, endogenous CNTF levels are elevated for up to 2 weeks, providing the transient activation of STAT3, which is important for antiapoptotic signaling (Table 1). Thus, this endogenous neurotrophic response is not sufficient to protect the injured RGC. However, administration of exogenous CNTF significantly extends the activation time of STAT3 (Ji et al., 2004). The CNTF concentration is decreased in the aqueous humor, lacrimal fluid and blood serum in patients with primary open–angle glaucoma (POAG); interestingly, the lowest levels of this neurotrophic factor positively correlate with a more advanced state of the disease (Shpak et al., 2017; Table 1). Interestingly, the NT-501 ECT (encapsulated cell therapy) implant, a device containing a genetically modified CNTF-secreting cell line, is under evaluation in 60 POAG patients enrolled in an ongoing randomized, sham controlled, masked phase II clinical study (NCT02862938) (Thanos et al., 2004; Barar et al., 2016; Table 2 and Figure 3).

### Vascular Endothelial Growth Factor A (VEGF-A)

Vascular endothelial growth factor A belongs to a superfamily including VEGF-B, -C, -D, and placental growth factor (PLGF). Among these members, the VEGF-A family is the most potent inducer of new vessel formation; more specifically, the pro-angiogenic VEGF_165_a isoform has a prominent role in neovascularization, and vessels permeability-related eye diseases, such as age-related macular degeneration (AMD) and diabetic retinopathy (DR) (Ferrara et al., 2003; Bandello et al., 2013; Amadio et al., 2016a). Beside the effects on vasculature, the subject of a large number of research studies and reviews, VEGF-A, in particular the VEGF_121_ and VEGF_165_ isoforms, also exhibits neurotrophic and neuroprotective activities. It has been reported that in normal conditions, VEGF_165_b is the predominant isoform in human eye tissues and fluids; it is downregulated in the vitreous fluid of DR patients, where a switch in VEGF splicing from anti- to pro-angiogenic isoforms (from VEGF_165_b to VEGF_165_a) seems to occur (Foxton et al., 2013). As with the pro-angiogenic VEGF_165_a, the anti-angiogenic VEGF_165_b exerts neuroprotective effects through VEGFR-2, p44/42 MAPK activation, and caspase-3 inhibition, but unlike VEGF_165_a, it does not involve either p38 MAPK or PI3K (Zachary, 2005; Beazley-Long et al., 2013). Interestingly, VEGF endogenous expression has been confirmed in RGCs and amacrine cells in the human retina, where it participates in RGC neuroprotection mechanisms (Famiglietti et al., 2003; Saint-Geniez et al., 2008; Beazley-Long et al., 2013; Foxton et al., 2013). This evidence suggests that pan-VEGF agents, although rapidly and effectively inhibiting neovascularization, vascular leakage, and other pathological changes in AMD or DR patients, exert detrimental effects in the long-term due to a loss of VEGF physiology at the ocular level (Amadio et al., 2016a; Gemenetzi et al., 2017; Fogli et al., 2018). Importantly, anti-VEGF therapies primarily targeting neovascularization result in thinning of RNFL and lack VEGF-A-mediated neuroprotection (Brar et al., 2010; Lv et al., 2014; Lee J.M. et al., 2017; Lee W.J. et al., 2017).

### Endogenous Growth Hormone

Growth hormone (GH) is a mitogenic peptide synthetized and released by somatotrophic cells in the pituitary gland. In addition, GH can be synthetized and released by neurons within the CNS, where it participates in stress-response and neuroprotection (Martínez-Moreno et al., 2018). In the human retina, GH and GH receptors (GHR) are expressed in RGCs, where the existence of RGC autocrine regulation by GH has been hypothesized (Sanders et al., 2006, 2009; Harvey et al., 2009). In their study, Sanders et al. (2006) demonstrated that following retinal insults, apoptotic RGCs coincided with GH-negative cells, while GH-expressing RGCs survived longer times. Further investigations in chick embryos led to the conclusion that GH neuroprotection is likely ascribed to anti-apoptotic effects mediated by tyrosine kinase receptors, Akt phosphorylation, *Bax* gene regulation and caspase-3 inhibition (Sanders et al., 2006).

## Factors Regulating the Redox Status

### Oxidative Stress Defense

In recent years, oxidative stress and mitochondrial dysfunction have been indicated as potential causes of glaucomatous neurodegeneration. In this multi-factorial disease, both the anterior and posterior segments of the eye, specifically the trabecular meshwork (TM)/endothelium and the inner retina/RGC, can be affected by oxidative damage, finally leading to visual pathway alteration in a series of closely linked events that needs to be fully elucidated. An increasing body of evidence suggests that the phenomena occurring in the anterior chamber, such as the oxidative stress-induced functional alterations of the TM, lead to defects in the posterior segment and are considered as the background of the pathogenesis of glaucoma – particularly, but not exclusively, high-tension glaucoma (Saccà et al., 2016). In glaucoma, the cellular and molecular mechanisms leading to cell death in the TM and RGCs show high similarities; cell loss is the consequence of apoptosis triggered by oxidative stress (Saccà et al., 2016). The maintenance of a balance between oxidative species production and clearance is particularly critical for the eye’s health. Oxidative stress occurs when concentrations of ROS rise above physiologic range with no adequate increase in the activity and/or levels of antioxidant defenses, what leads to damage of cellular components (proteins, nucleic acids, and lipids) by oxidation and eventually to cell degeneration/loss (Halliwell and Gutteridge, 1999). In physiologic conditions, various antioxidant and detoxifying factors at the ocular level, act in concert removing effectively ROS; however, harmful stimuli can lead to changes/malfunctioning in one or more antioxidant defense systems, what affects the global redox balance and finally contributes to pathological conditions. As already mentioned for neurotrophins, several lines of evidence indicate that the protein levels and enzymatic activities of antioxidant defenses in the aqueous humor (AH) are significantly altered in glaucoma. It was shown that global antioxidant potential level in the AH of glaucoma patients was lower than the mean of control cataract group (Ferreira et al., 2004), suggesting that a chronic exposure to ROS contributes in glaucoma to the progressive loss of TM cellularity and subsequent change of redox balance (Alvarado et al., 1984). At the systemic level, patients with POAG presented decreased total plasma antioxidant capacity compared to healthy subjects (Abu-Amero et al., 2013), what is in agreement with previous findings of Erdurmuş et al. (2011) in the serum of patients with POAG and pseudoexfoliative glaucoma (PEG). Contrary, primary angle closure glaucoma (PACG) patients and control subjects showed comparable total serum antioxidant levels (Abu-Amero et al., 2014a).

### Endogenous Antioxidant Factors

Among antioxidants and detoxifying factors, glutathione (GSH) and the enzymes in the GSH pathway (GSH peroxidase, reductase, and transferase), superoxide dismutase (SOD), and catalase, are the most studied in relation to RGCs.

Glutathione is a low-molecular weight detoxifying molecule present in all mammalian tissues. In its reduced state, GSH is considered the master free radical scavenger, especially at the mitochondrial level, being used by GSH peroxidase (GPx) to inactivate hydrogen peroxide. This reaction leads to the formation of oxidized GSH, or GSSG; new GSH can be restored by GSH reductase in the presence of NADPH. GSH can also be conjugated to harmful xenobiotic substances by GSH transferase (GST), with the aim of detoxification. Blood GSH levels inversely correlate with aging in healthy subjects but not in glaucoma patients; however, independently of age, glaucoma patients displayed lower GSH content than the healthy individuals (Gherghel et al., 2005).

SOD converts the highly cytotoxic superoxide anion into hydrogen peroxide, and it is present in all eye structures. Three human SOD isoforms (the cytosolic Cu-ZnSOD, also named SOD1; the mitochondrial MnSOD, or SOD2; and the extracellular EC-SOD, or SOD3) have been detected in AH (Behndig et al., 1998). In this fluid, decreased expression of SOD1/2 as well as of GST were observed in glaucoma patients in comparison to cataract control group (Bagnis et al., 2012). Conversely in serum, higher SOD1/2 levels were reported in glaucoma patients than controls (Erdurmuş et al., 2011). Increased SOD, as well as GPx activity, has been found in AH of glaucoma patients compared to cataract patients (Ferreira et al., 2004; Goyal et al., 2014), as a possible compensating response to higher ROS levels. More recently, impairment in total serum SOD activity was detected in the glaucoma group, displaying specifically lower MnSOD levels than controls (Rokicki et al., 2017). In this study, glaucoma patients presented with a rise in the total oxidative status but not in the total antioxidant capacity.

Catalase (CAT) brakes hydrogen peroxide to water and oxygen. Some studies aimed to evaluate CAT in either AH or serum, or both, reported no significant differences in this enzyme (either levels or activity) between glaucoma and control subjects (Ferreira et al., 2004; Ghanem et al., 2010; Goyal et al., 2014), while others demonstrated an impairment in CAT in glaucoma (Majsterek et al., 2011). A significant heterogeneity in the methods used to detect enzymatic activity can be found among the literature studies. Some of them measured the “total” enzymatic activity of the SOD family, others the activity of the specific isoforms (e.g., SOD1 and SOD2). In addition, the diversity of glaucoma types evaluated in these studies might be an additional source of variability and a critical point for the interpretation of the findings. A recent reviews and meta-analysis provide a more homogeneous picture of the local and systemic alterations in glaucoma patients, revealed an overall increase in oxidative stress markers in chronic glaucoma, in both the serum and the AH. Despite a general decrease in antioxidant markers in the serum, SOD and GPx increased in the AH, likely as a protective response in the eye against oxidative stress (Benoist d’Azy et al., 2016). A compensatory increase in SOD and GPx activity in association with increased oxidative processes was also found in the brains of the mouse model for glaucoma (Ferreira et al., 2013), supporting idea about the close link and mutual influence existing between the eye and brain.

In general, discrepancies between expression level and enzymatic activity are not infrequent, and they are not limited to compensatory mechanisms, where the decreased activity of a given enzyme (due to an age-related impairment of the biological system or any more specific cause) may be counterbalanced by an increase in its expression. Likewise, high concentration of an enzyme does not necessarily mean high activity. Gene polymorphisms of antioxidant enzymes may indeed be responsible for changes in their activity, possibly resulting in consecutive effects of glaucoma-induced oxidative damage.

The most common and best studied SNP of *SOD2* is the rs4880 allele. The rs4880 (C47T) (C) and (T) alleles give rise to either alanine (Ala) or valine (Val), respectively, at codon 16, located within the mitochondrial targeting sequence of SOD. There are conflicts in the literature over the effect of this SNP. Val (T) was associated with a less efficient transport of SOD2 in the mitochondria and a lower enzyme activity compared to Ala (C) (Sutton et al., 2005; Tian et al., 2011). However, in another study on healthy human erythrocytes isolated from Asian or Caucasian volunteers, SOD2 activity was 33% higher in (T;T) or (C;T) individuals compared to (C;C) subjects (Bastaki et al., 2006). The same authors reported significant variation of allele frequencies between ethnicities; SOD2 enzyme activity was also shown to be higher (+15%) in females than males. The differences between the lowest and highest levels of medium enzymatic activity were relevant not only for SOD2 but also for GSx and CAT (56-fold, 6-fold, and 8-fold, respectively). We can conclude that antioxidant enzyme activities show a high inter-individual variability that may be related to genetic polymorphisms and that gender and ethnicity probably contribute to discrepancies among the various studies. The possible role of the C47T SOD2 mutation in normal-tension glaucoma pathogenesis was investigated in a German population and no association was found (Wolf et al., 2009). Similar findings were obtained in Saudi patients with POAG; however, on the basis of the study results, the authors suggested that the C47T SOD2 mutation can be a risk factor for various clinical indices, such as high IOP and severe clinical course (Abu-Amero et al., 2014b). The possible link between SOD1 35A/C and the risk of POAG was studied in a Polish population and no association was found (Malinowska et al., 2016). The same study found instead that the C/T genotype of both GPx Pro198 Leu and CAT -262C/T confers major risks to developing POAG.

### Antioxidant Therapies for RGC Degeneration

Based on these studies, several antioxidant agents, as well as molecules indirectly affecting factors/pathways related to redox response (such as those blocking glutamate excitotoxicity-induced oxidative stress), have been shown to provide RGC protection in *in vivo* models, and may be of interest for glaucoma treatment and/or prevention (Song et al., 2015; Nuzzi and Tridico, 2017). The authors are aware that changes of the endogenous antioxidant factor levels may represent only one of the protective effects, and that other underlying molecular mechanisms may be responsible of the RGC loss prevention provided by these agents; however, a thorough discussion and critical interpretation of the cited evidence are beyond the scope of this review.

It was reported that retinal GSH decreases due to ischemia/reperfusion (I/R) injury were counteracted by subcutaneous Vitamin E treatment in guinea pigs (Aydemir et al., 2004); similarly, subcutaneous administration of Vitamin E, as well as carotenoid derivative – Lutein, reversed the decreased GSH levels in rat retinas with I/R injuries (Dilsiz et al., 2006). GSH decrease in the retinas of glaucomatous mice was prevented by oral administration of alpha-Luminol GVT^®^, a compound endowed with antioxidant and anti-inflammatory properties (Gionfriddo et al., 2009). In rat retinas with I/R injury, intraperitoneal injection of Methane increased both the antioxidant enzyme activities (SOD, CAT, GPx) and anti-apoptotic gene (*Bcl2*) expression, ultimately decreasing RGC loss, total retinal layer thinning, and visual dysfunction (Liu et al., 2016). Intraperitoneal injection of Crocin, a pharmacologically active component of *Crocus sativus L.* (saffron), increased GSH levels and total SOD activity, decreased ROS and counteracted RGC loss in I/R injury (Chen L. et al., 2015). It was also reported that a diet supplemented with alpha-lipoic acid (ALA), either alone (Inman et al., 2013) or in association with SOD (Nebbioso et al., 2013), was able to protect RGCs in the presence of ocular ischemic and pressure stress. Transgenic SOD1-overexpressing mice showed accelerated RGCs death after ON injury and showed an enhancement of RGC survival when systemically injected with alpha2-adrenoreceptor agonist brimonidine (Levkovitch-Verbin et al., 2000). In animal models of rat glaucoma and rabbit retinal NMDA excitotoxicity, it has been demonstrated that brimonidine provides neuroprotection by modulating NMDA receptor function through postsynaptic alpha2-adrenoreceptors in RGCs; the alpha2-mediated brimonidine effect leads to a reduction of intracellular cAMP production, the latter being responsible for NMDA activation in RGCs (Dong et al., 2008). *In vivo* evidence confirmed that topical treatment with brimonidine decreased retinal damage induced by ocular hypertension and showed that analogous results were obtained by intraperitoneal administration of N-acetyl cysteine (Ozdemir et al., 2009). Brimonidine was compared to timolol in the Low-pressure Glaucoma Treatment Study – LoGTS – with the aim of evaluating if either treatment protected the optic nerve and prevented vision loss in adults (Sena and Lindsley, 2017). After 4 years, the brimonidine group showed less progressive loss of the visual field than the timolol group; however, since many people dropped out of the study, especially from the brimonidine group, and the authors did not report data for visual acuity, the interpretation of the results was difficult and evidence of the real effectiveness of brimonidine as a neuroprotective agent in glaucoma was not provided.

In the panorama of gene therapy, intravitreal, adeno-associated. virus-mediated pretreatment with SOD2 attenuated oxidative stress and reduced mitochondrial dysfunction in RGCs and the ON in glaucoma (Jiang et al., 2016). In comparison with wild-type counterparts, RGC survival and up-regulation of antioxidant enzymes (such as SOD2, CAT, GPx), were observed in I/R retinas of mice overexpressing Frataxin, a mitochondrial protein serving important functions in iron homeostasis and oxidative stress responses (Schultz et al., 2016). Protection of RGCs was also obtained in a mouse model of partial ON-crush following oral administration of Persimmon leave (*Diospyros kaki*) extract; this extract rich in several bioactive compounds, exerts antioxidant and pro-survival activities by modulating the expression of defensive and apoptotic genes (by increasing SOD1, GST, GPx; or by decreasing PPAR, p53, respectively) (Ryul Ahn et al., 2017). Oral administration of Ginkgo biloba extract seems to provide beneficial effects on RGCs in a glaucoma animal model (Hirooka et al., 2004) and to ameliorate pre-existing visual field damage in some patients with normal-tension glaucoma (Quaranta et al., 2003; Lee et al., 2012). However, Guo et al. (2014) did not show a statistically significant difference in visual field outcomes after Ginkgo biloba supplementation in Chinese patients. These conflicting reports suggest that racial differences may also play an important role (Kang and Lin, 2018). In addition to antioxidant properties, Ginkgo biloba increases blood flow through vasodilation and reduces blood viscosity (Kang and Lin, 2018). The safety and efficacy of a combination of antioxidants and anti-inflammatory agents in OAG patients are currently under evaluation in a phase I clinical trial (NCT02984813) (Table 2).

*In vivo* evidence of neuroprotection, mainly in favor of RGCs, have been reported for other antioxidant/antiapoptotic agents, such as *Lycium Barbarum Lynn* extracts (Chan et al., 2007), Stanniocalcin-1 (Kim et al., 2013), Oryzanol (Panchal et al., 2017), Resveratrol and Riluzole, tested either alone or in combination in glaucoma models (Lindsey et al., 2015; Pirhan et al., 2016). Interestingly, evidence in a mouse model of normal-tension glaucoma showed that caloric restriction promotes retinal cell survival by increasing the expression of CAT and neurotrophic factors and by reducing global oxidative stress (Guo et al., 2016). Another antioxidant – taurine protects cells against oxidative stress by upregulating enzymes such as thioredoxin reductase, glutathione peroxidase and SOD. It is suggested that a decrease in retinal blood perfusion in glaucoma may reduce taurine uptake and cause degeneration of RGCs (Yildirim et al., 2007; Froger et al., 2012, 2013).

It is important to have different therapeutic approaches to counteract glaucoma progression, for instance some drugs currently used to control the disease, such as timolol and dorzolamide, display antioxidant properties, possibly contributing to their therapeutic effects (Izzotti et al., 2008; Sacca et al., 2011). Pronounced oxidative stress is a feature of mitochondrial optic neuropathies, including LHON (Man et al., 2002). In this group of inherited diseases, a genetic defect within RGC mitochondrial proteins results in a functional problems in mitochondrial oxidative phosphorylation and subsequent decrease of energetic substrates availability leads to RGC degeneration (Carelli et al., 2004). Supplementation of coenzyme Q_10_ analog idebenone enables electron transfer through complex I-III of the mitochondrial respiratory chain, reduces ROS production and energy disbalance and provides sufficient neuroprotection and RGC recovery in certain groups of LHON patients (El-Hattab et al., 2017; Yu-Wai-Man et al., 2017). It is worth mentioning that two phase IV clinical trials on idebenone (Raxone^®^) in LHON patients started in 2016 and are currently ongoing (NCT02774005).

### NF-E2-Related Factor 2 (Nrf2)

In the constellation of novel potential neuroprotective therapies, the modulation of expression of enzymes/proteins/factors may be a strategy to increase antioxidant defenses. In this regard, many antioxidant and detoxifying enzymes, such as GST, SOD, are regulated by neuroprotective NF-E2-related factor 2 (Nrf2), a transcription factor activated by oxidative stress and electrophilic molecules and whose relevance in many ocular diseases including glaucoma has been recently reviewed (Batliwala et al., 2017). Nrf2 deficiency is characterized by increased oxidative stress and neuronal degeneration and aggravates RGC loss in animals subjected to either ON damage (Himori et al., 2013) or I/R injury (Wei Y. et al., 2011).

Interestingly, among *in vivo* evidence for potential neuroprotective agents, the Nrf2 activator triterpenoid 1-(2-cyano-3-,12-dioxooleana-1,9(11)-dien-28-oyl) imidazole (CDDO-Im) reduced ON crush-induced RGC death in mice by upregulating the expression of antioxidant and phase II detoxifying enzymes (Himori et al., 2013). Similar findings were reported for CDDO-Im in mice subjected to I/R (Xu et al., 2015). Treatment with Trimetazidine, an anti-ischemic drug and metabolic modulator endowed with neuroprotective properties, attenuated retinal damage and RGC death in an acute glaucoma animal model by activating the Nrf2-pathway (Wan et al., 2017), further suggesting that pharmacologic induction of the Nrf2-pathway may represent a novel neuroprotective tool in retinal diseases. In agreement, the Nrf2 modulator monomethyl fumarate, the biologically active metabolite of dimethyl fumarate, displayed neuroprotective effects and decreased neuronal cell loss in the ganglion cell layer of mouse retinas after I/R injuries (Cho et al., 2015). New achievements in gene therapy allowed to spatio-temporal regulation of Nrf2 expression by specifically targeting RGCs at risk of degeneration in murine ON injury, thus preventing death of stressed cells and limiting undesired off-target effects on healthy neurons (Fujita et al., 2017).

## Factors and Pathways Regulating Apoptosis/Cell Death and Neuroinflammation

### P53

One of the important regulatory proteins involved in apoptosis of RGCs is the tumor suppressor protein p53. This protein is a transcription factor which upregulates the expression of the proapoptotic gene *Bax* and downregulates the expression of the antiapoptotic gene *Bcl2* (Daugherty et al., 2009; Fan et al., 2010). It is suggested that *p53* gene polymorphisms may be involved in POAG pathogenesis. It has been shown that the properties of p53 change depending on the residue occupying position 72 in the peptide chain. In particular, the codon 72 polymorphism involves a proline (Pro) to arginine (Arg) amino acid substitution at position 72 (Pro72Arg). The Arg72 isoform more efficiently induces apoptosis, while Pro72 shows greater ability to arrest the cell cycle in response to DNA damage (Lin et al., 2002; Neamatzadeh et al., 2015; Gohari et al., 2016). However, some of the abovementioned studies concerning the association between the codon 72 polymorphism and POAG are in disagreement, suggesting that ethnic differences may affect genetic predisposition to this disease (Neamatzadeh et al., 2015; Gohari et al., 2016). In particular, some evidence indicates that the p53 codon 72 polymorphism may be associated with increased risk of POAG in Asian but not in Caucasian populations (Guo et al., 2012; Neamatzadeh et al., 2015; Gohari et al., 2016), while others have not confirmed these results (Mabuchi et al., 2009). Furthermore, some reports suggest that the Arg72Pro polymorphism of the *TP53* gene may be related to progression of POAG rather than with risk of occurrence of this disease (Nowak et al., 2014).

### Heat Shock Proteins

Heat shock proteins (Hsp) represent a family of proteins playing the role of molecular chaperones – regulating proper macromolecule turnover in the cytosol (Ikwegbue et al., 2017). Hsp are involved in oxidative stress defense, inhibition of proinflammatory cytokines and inhibition of apoptotic pathways. They supervise protein folding and unfolding, as well as degradation of irreparably misfolded proteins (Srivastava, 2002; Kaarniranta et al., 2009; Fontaine et al., 2016; Kampinga and Bergink, 2016). Regarding RGCs, the function of Hsp70 and Hsp27 are the most widely documented (Chidlow et al., 2014; Piri et al., 2016). Induction of Hsp can inhibit apoptosis both directly – via suppression of proapoptotic factors, i.e., p53 and a wide range of Bcl2 family members, including Bax, Bid, Akt and Apaf-1, – and indirectly – by suppressing proinflammatory cytokines, i.e., IL17, IL1β, TNFα, or IL-8 and inhibiting oxidative stress (Jacquier-Sarlin et al., 1994; Ravagnan et al., 2001; Takayama et al., 2003). Fatal cellular effects of oxidative stress alleviated by Hsp include repair of misfolded proteins, prevention of protein aggregation, and reduction of ROS-dependent genotoxicity (Jacquier-Sarlin et al., 1994; Mayer and Bukau, 2005). In humans, *Hsp70* gene polymorphism is related to a higher risk of POAG development, confirming the relevance of this Hsp in endogenous neuroprotection of RGCs (Nowak et al., 2015).

### The BCL-2 Family

A major role in the apoptotic process of RGCs during glaucoma is played by members of the BCL-2 family. In glaucoma, the decreased expression of the pro-survival bcl-2 and bcl-xl genes is accompanied by an increase in pro-apoptotic bax and bad gene expression. The proapoptotic BCL-2 family of proteins promotes the release of cytochrome c from the mitochondrial intermembrane space into the cytoplasm. Cytochrome c binding to apoptosis inducing factor-1 (Apaf-1) and procaspase-9 activates caspase-9 and then caspase-3 and -7 causing apoptosis. Bcl-2 inhibits this pathway by blocking the activation of bax and bak (Levkovitch-Verbin, 2015; Gauthier and Liu, 2017). Overexpression of bcl-2 prevents RGC loss in a rat axotomy model (Kretz et al., 2004; Malik et al., 2005). Moreover, changes in expression of transcription factors such as immediate early genes (IEGs) which regulate the transcription of bax and bcl-2 genes, are observed in various experimental glaucoma models and the changes in the expression of these genes can be associated with both RGC apoptosis and survival (Kwong and Caprioli, 2006; Levkovitch-Verbin et al., 2007; Levkovitch-Verbin, 2015). In addition, overexpression of bcl-2 leads to an increase in the cellular content of GSH, whose involvement in the antioxidant defense system has already been mentioned (Moon et al., 2013). Bax deficiency completely prevented RGC death in DBA/2J mice because the RGC death pathway is Bax-dependent, whereas the axonal degeneration pathway is Bax-independent. Additionally, Bax deficiency delays IOP elevation in glaucoma (Libby et al., 2005). Furthermore, activation of caspases 3, 6, 7, and 9 may be endogenously counteracted by inhibitor of apoptosis proteins (IAPs) (Levkovitch-Verbin, 2015; Gauthier and Liu, 2017). It is suggested that members of the IAP family (IAP_1_ and XIAP) may represent endogenous neuroprotective mechanisms that are activated in the retina in response to elevated IOP. Expression of IAPs decreases in glaucomatous retinas and correlates with age, suggesting that an impairment in the IAP system increases the retina’s vulnerability to elevated IOP (Levkovitch-Verbin et al., 2013). It is worth mentioning that an siRNA against caspase 2 mRNA, QPI-1007 (Quark Pharmaceuticals) received orphan designation by FDA for optic neuropathy and it is currently under evaluation in phase II clinical trial for glaucoma (NCT01965106) (Figure 3).

### RNA-Binding Protein HuR

The expression of defense genes can also be modulated by post-transcriptional mechanisms, such as RNA-binding proteins – RBPs – and microRNAs, which allow punctual, rapid and localized changes in gene product levels. This control of gene expression is particularly relevant in neurons, whose projections can extend for long distances from the nucleus. A derangement in these mechanisms may thus seriously endanger the physiological cellular response to changing external conditions, representing a critical point of failure in endogenous neuroprotection. Changes in RBPs have been associated with various neurodegenerative diseases (Pascale and Govoni, 2012; De Conti et al., 2017); in particular, the RBP HuR/ELAVL1 regulates the expression of hundreds of genes, including stress response proteins (such as SOD1, p53, Hsp70) (Mazan-Mamczarz et al., 2003; Amadio et al., 2008; Milani et al., 2013) and pro- and anti-inflammatory factors (Nabors et al., 2001), playing a key role in cell survival/apoptosis under stress conditions (Lebedeva et al., 2011). Under endogenous or external stimuli (growth factors, inflammatory stimuli, hypoxia, oxidative stress and many others), HuR protein is activated and moves from the nucleus to the cytoplasm, where it favors the stability and/or translation of target RNAs (Abdelmohsen et al., 2008; Doller et al., 2008; Zucal et al., 2015). Lately, the involvement of HuR in mechanisms underlying ocular pathologies has received increasing interest (Amadio et al., 2010, 2012, 2016b; Joseph et al., 2012; Viiri et al., 2013). Relevant for our context, *in vitro* studies showed that HuR was activated by oxidative stress in TM cells (Mochizuki et al., 2012). More recently, *in vivo* evidence by our group revealed that changes in HuR subcellular localization (i.e., nuclear-cytoplasmic shuttling) within RGCs occurred at early times after IOP induction in an animal model of glaucoma; these effects were followed at longer times by a progressive decrease of cytoplasmic HuR levels, including the expression of proteins essential for cell homeostasis (p53, Hsp70) and likely contributes to chronic IOP-induced RGC degeneration (Smedowski et al., 2018). Similar alterations in HuR content and subcellular localization were found in human POAG samples, in support of the involvement of HuR in glaucoma (Smedowski et al., 2018). A relevant role of HuR in neuroprotection was also described in brain neurons (Skliris et al., 2015). The potential of HuR as a new pharmacological target is shown by an increasing interest in medicinal chemistry by the field (for a review, see Nasti et al., 2017). There is also some evidence of altered microRNA expression in the AH of glaucoma patients (Jayaram et al., 2017); however, the role of microRNAs, as well as of RBPs and, more generally, of post-transcriptional mechanisms controlling gene expression in glaucoma, needs to be further explored.

### Metallothioneins

Metallothioneins I-IV (MT I-IV) are a family of four metalloproteins that have multiple activities, such as antioxidant (ROS scavenging), regulation of metal homeostasis (mostly Zn, potential heavy metals scavenger), and transcription factors synthesis (MT are a source of Zn for enzymes). MT I, II and III have been documented to have a pivotal role in stress response in neurons (Suemori et al., 2006; Pedersen et al., 2009). In healthy retinas MT I/II and III are expressed mostly in RGC axons (retinal nerve fiber layer) and the inner plexiform layer (Suemori et al., 2006). Similarly, isoforms MT I and III have been detected in proteomic analyses of ON homogenates (Smedowski et al., 2018). In stress conditions, expression of MT has been shown to increase within RNFL and RGC bodies, suggesting its role as an endogenous neuroprotective factor (Suemori et al., 2006). Beside their scavenging properties MT play an important antiapoptotic role (through inhibition of p53, caspase 1, 3, 9 and cytochrome c leakage) and anti-inflammatory activity (by decreasing expression of proinflammatory cytokines, and inhibiting macrophages differentiation). Finally, MT have a strong impact on neuroregeneration and axonal regrowth via the megalin (LRP2) receptor, by increasing the expression of growth factors (NGF, BDNF, GDNF, NT, VEGF) in CNS (Pedersen et al., 2009). This phenomenon was also found and confirmed in RGCs (Fitzgerald et al., 2007).

### Tumor Necrosis Factor Alpha (TNFα) and Neuroinflammation

Neuroinflammation is one of the processes involved in the neurodegeneration, also in the retina and the optic nerve (Soto and Howell, 2014; Williams et al., 2017). TNFα is considered the critical regulator of neuroinflammation both in the retina and optic nerve, which together with its target receptors TNFR1 and TNFR2 may regulate homeostasis as well as pathophysiological processes. The involvement of TNFα gene and protein alteration in retina has been reported in animal model of glaucoma and human primary open angle glaucoma (Yang et al., 2011; Xin et al., 2013; Wilson et al., 2015). In retina and optic nerve, TNFα is rapidly release from glial cells (i.e., microglia, Müller cells) as a response to cell-stress inducing factors, like increased intraocular pressure (Cueva Vargas et al., 2015). It has been shown that soluble TNFα fraction is responsible for detrimental pro-inflammatory and pro-degenerative effects in RGCs and is related to binding with TNFR1, while stimulation of TNFR2 can exhibit protective effects (Cueva Vargas et al., 2015). The molecular pathways of the TNFα-depended neurodegeneration relay on increasing of Ca^2+^ permeability of AMPA receptor (AMPAR) and accumulation of Ca^2+^ in cytosol, mitochondrial damage and ROS production (Arundine and Tymianski, 2003; Crish and Calkins, 2011). On the other hand, binding of TNFα to TNFR1 can directly mediate apoptotic response via Fas-Associated Death Domain (FADD) and caspase-3/caspase-8 pathways activation (Agarwal and Agarwal, 2012). Moreover, TNFα by binding to TNFR2 can also trigger the activation of survival signals through the stimulation of a transcription factor NF-KB, which inhibit apoptosis. Besides TNFα induces another protection mechanism represented by HSPs (Tezel, 2008). From the excitotoxic point of view, overexpression of TNFα favors the activity of excitatory synapses, inhibits uptake of glutamate by astrocytes and increases release of glutamate from microglia, which all leads to overload of extracellular space with this mediator and results in self-perpetuating excitotoxic insult (Olmos and Lladó, 2014). Despite of growing evidences of involvement of TNFα pathways in RGC degeneration, there is a clear lack of targeted anti-TNFα, neuroprotective therapies. The promising attempts were done using the inhibitors of soluble TNFα fraction, achieving alleviation of RGC damage in ocular hypertension model (Cueva Vargas et al., 2015). Applying of non-selective anti-TNFα agent (etanercept) suppressed microglial activation and provided optic nerve axons neuroprotection in the rat ocular hypertension model (Roh et al., 2012). Interestingly, there are several known TNFα cross-interacting pathways, with described neuroprotective factors. ELAVL1/HuR protein regulates the expression of TNFα, as well as other pro- and anti-inflammatory cytokines, like IL-1, IL-6, IL-8, IL-10, participating in cellular homeostasis (Smedowski et al., 2018). TNFα expression is also regulated by erythropoietin, however, the mechanism of this cross-pathway depends on the way of inflammatory process activation (Hines-Beard et al., 2016). In the inflammation induced by lipopolysaccharide (LPS), the release of TNFα by microglial cells was proved to be controlled by systemic erythropoietin gene delivery (Yazihan et al., 2008). This approach was ineffective in case of neuroinflammation associated with optic neuropathy, where macroglial cells are the major source of TNFα (Tezel and Wax, 2000). In the last case, local erythropoietin gene delivery (i.e., intravitreal) seems to be more efficient option (Hines-Beard et al., 2016). The neuroprotective effects mediated by down-streaming of TNFα and other proinflammatory cytokines was also reported for endocannabinoids and metallothioneins (Helal et al., 2009; Krishnan and Chatterjee, 2012).

### Erythropoietin and Erythropoietin Receptor

Erythropoietin (EPO) is a hematopoietic cytokine known as a stimulant of erythropoiesis in response to hypoxia (Erslev and Caro, 1986; Shih et al., 2018). However, more recently, in addition to bone marrow, the receptor for EPO (EPO-R) has been identified in multiple tissues, including the retina and, more specifically, in RGCs (Becerra and Amaral, 2002; Shah et al., 2009). Stimulation of neuronal EPO-R plays an important role in response to stress and injury and prevents neuronal apoptosis by alleviation of effects of hypoxia, glutamate excitotoxicity and growth-factors deprivation (Junk et al., 2002). In their study, Junk et al. (2002) proved the existence of an endogenous neuroprotective system consisting of EPO/EPO-R that participates in stress response in retinal ischemia/reperfusion. Fu et al. (2008) studied specific localization of endogenous EPO/EPO-R expression and demonstrated that in healthy conditions, endogenous EPO is expressed among others in the RNFL (RGC axons) and EPO-R in RGC bodies; the expression of both the cytokine and the receptor increases in the rat retina following a glaucoma-mimicking injury. In a DR model, it has been suggested that the molecular basis of EPO activity in RGC is related to its antioxidant action – by increasing the activities of SOD, GSH-Px, and CAT and by preventing the generation of pro-apoptotic signals what improves RGC survival (Wang Y. et al., 2014). Protective EPO activity has also been demonstrated in other models of RGC death, i.e., NMDA excitotoxicity, neurotrophic factors deprivation, inflammatory insults, ON crush and glaucoma (Zhong et al., 2007, 2008; Rong et al., 2011; Chang et al., 2013). In RGC axotomy, EPO-mediated neuroprotection can be related to extracellular signal-regulated kinases (Kilic et al., 2005). Beside neuroprotection, stimulation of EPO-R can also induce axonal regeneration within RGCs by upregulating growth associated protein-43 and downregulating RhoA and ROCK (Tan et al., 2012a,b). It is worth remembering that RhoA/ROCK signaling modulates AH outflow; ROCK inhibitors lower intraocular pressure (IOP) via a direct effect on TM and Schlemm’s canal, and they are currently under clinical evaluation in glaucoma (Wang and Chang, 2014). Interestingly, it has been reported that topical administration of a ROCK inhibitor promotes RGC survival and axon regeneration after ON injury *in vivo* (Shaw et al., 2017), suggesting that the beneficial effects of this class of inhibitors may go beyond their action on IOP.

The role of EPO in the eye, its potential for treatment of ocular disorders, and some clinical trials on EPO and EPO derivatives in various ocular disorders other than glaucoma, have been reviewed by Shirley Ding et al. (2016). However, to our knowledge, there are no ongoing clinical trials of EPO therapy for glaucoma.

### Endocannabinoids

Endocannabinoids (eCBs, i.e., anandamide and 2-arachidonoylglycerol) are physiological ligands for the cannabinoid receptors CB1 and CB2. They represent arachidonate-based retrograde neurotransmitters playing a relevant role in a variety of physiological processes in the central and peripheral nervous systems, including neuroprotection and synaptic plasticity (Xu and Chen, 2015). In the eye, beside the effect of lowering the intraocular pressure (i.e., anandamide), the eCBs system in RGCs modulates neurotransmitter release, enhances processing and integration of visual signals by interacting with the TRPV1 channel and Ca^2+^ and K^+^ ion channels (Pate et al., 1995; Cécyre et al., 2013; Miraucourt et al., 2016; Jo et al., 2017). Nucci et al. proved that the rat retina has a complete and functional endocannabinoid system involving synthesis, transport, hydrolysis and binding of anandamide (Nucci et al., 2007; Schwitzer et al., 2016). Moreover, a decrease of anandamide in retinal ischemia may be involved in RGC loss (Nucci et al., 2007). Although both CB1 and CB2 receptors are present in the retina, the neuroprotective activity of eCBs has been investigated in various models of RGC pathology and seems to be related to CB1 receptor (Kokona et al., 2016). In I/R and excitotoxic models of retinal degeneration, stimulation of the CB1 receptor provided neuroprotective effects in different retinal neuron populations (Kokona et al., 2016). The effect was related to reduction of excitotoxic insult via modulation of glutamate release and activation of antiapoptotic pathways involving PI3K/Akt and MEK/ERK1/2 (Wartmann et al., 1995; Gómez del Pulgar et al., 2000; Karanian, 2005; Molina-Holgado et al., 2005; Ozaita et al., 2007). In a glaucoma model, activation of CB1 and TRPV1 receptors by the agonist methanandamide, provided RGC neuroprotection and created a possible new target for glaucoma therapy (Nucci et al., 2007). In the streptozotocin-induced DR model, CB treatment provided better preservation of the blood-retina barrier and anti-inflammatory effects of downstream proinflammatory cytokines, i.e., TNFα (El-Remessy et al., 2006).

### Neuroglobin

Neuroglobin (Ngb) is an oxygen-binding heme protein containing iron, represents the neuronal counterpart of blood hemoglobin. Ngb plays a crucial role in endogenous neuroprotection in CNS and RGCs (Yu et al., 2012). Ngb can reversibly bind oxygen; thus, it is associated with mitochondrial metabolism and the respiratory chain; Ngb can also supply oxygen to neurons (Lechauve et al., 2012). Due to its high oxygen affinity, Ngb can scavenge ROS, modulating NO-related processes and inducing stress-response in cells due to hypoxia (Wei X. et al., 2011). In RGC pathology, i.e., retinal ischemia and glaucoma, Ngb participates in the stress-response by having an impact on RGC integrity and survival by preventing mitochondrial protein damage and energy failure due to respiratory chain impairment (Wei X. et al., 2011; Cwerman-Thibault et al., 2017). RGC degeneration may be linked with decreased Ngb content, and supplementation with exogenous Ngb is shown to alleviate RGC death and even to induce axonal outgrowth after ON crush (Sugitani et al., 2017). In DBA2J mice, where glaucomatous-like ON pathology is directly linked with failure of mitochondrial bioenergetics and decreased Ngb content, exogenous delivery of Ngb can slow down progression of RGC degeneration or even reverse it, which brings up a promising application in mitochondrial ON diseases, i.e., LHON (Cwerman-Thibault et al., 2017).

### Estrogens and Their Receptors

Estrogens are steroid hormones primarily related to the reproductive, skeletal and cardiovascular systems (Munaut et al., 2001). Estrogen receptors α and β (ER α and β) are expressed within the ocular elements, including the retina, and especially in RGCs (Munaut et al., 2001). The function of estrogen signaling has been proven to be related to cataractogenesis, dry eye syndrome and AMD (Gupta et al., 2005). Recent studies also showed involvement of ER β receptors in the endogenous neuroprotection of axotomized RGCs via activation of the ERK-c-Fos pathway (Nakazawa et al., 2006), in glaucomatous neurodegeneration via Akt/CREB/thioredoxin-1, MAPK/NF-kappaB and inhibition of IL-18 (Zhou et al., 2007; Russo et al., 2008; Prokai-Tatrai et al., 2013). In ischemic optic neuropathy, estrogens prevent RGC degeneration if applied before the insult, with no effects of treatment post-insult (Bernstein et al., 2007). Estrogens can prevent the effects of oxidative insult in retinal neurons by activation of PI3K/Akt signaling and exert mitochondrial protection associated with the attenuation of the proapoptotic Bax gene (Li et al., 2013; Hao et al., 2014). There is evidence that topical delivery of 17β-estradiol can prevent RGC death in a glaucoma model in rats (Prokai-Tatrai et al., 2013). In another study, Vajaranant and Pasquale (2013) showed that estrogen deficiency associated with aging, accelerated optic nerve dysfunction. There is also clinical evidence that postmenopausal estrogen supplementation in women can significantly reduce the risk of POAG development (Newman-Casey et al., 2014) and that ER2 receptor polymorphism can be associated with increased risk of POAG in men (de Voogd et al., 2008).

### Autophagy – Neuroprotective Aspects

Autophagy is a cellular clearing system for scavenging misfolded proteins, lipids and other cell components; it prevents protein aggregation and maintains proper organelle turnover, thus assuring cell survival. Autophagy prevents dysfunction of cellular components which may appear due to oxidative insults and increased mitochondrial membrane permeability (Davis et al., 2016). In RGCs, the autophagy process has been studied in ocular hypertension models, retinal and ON ischemia, and transection or ON crush (Lin and Kuang, 2014). In glaucoma, opposite changes in the autophagy process can be observed in RGCs when compared to the ON; in particular, an increase in autophagy is observed in the cell soma/RGC, and autophagy is impaired in the axons/optic nerve (Deng et al., 2013; Kleesattel et al., 2015). The significance of this process in RGC biology has not been fully determined yet, and at present it is still unclear whether induction or inhibition leads to beneficial outcomes (Park et al., 2012; Su et al., 2014; Koch and Lingor, 2016). Accordingly to this, the effects of autophagy activation may also depend on the cellular compartment in which it takes place; i.e., in glaucoma, autophagy may be initially activated in RGC dendrites and axons, which possibly has protective outcomes, but under the chronic increase of intraocular pressure, autophagosomes appear in the cytoplasm of the cell body, mediating cell death (Lin and Kuang, 2014). Interestingly, some of the abovementioned factors have been related to autophagy, suggesting it plays an important role in maintaining RGC homeostasis.

### Endoplasmic Reticulum Stress Modulation

Different damaging factors may affect intracellular pathways by impairing correct proteins turnover, resulting in accumulation of unfolded and misfolded proteins in the lumen of endoplasmic reticulum (ER). The cell-stress response to this event, known as the unfolded protein response (UPR) activates cascade of reactions and pathways leading to cell death by apoptosis (Xu et al., 2005). Recently, the UPR has been postulated to be involved in the pathogenesis of neurodegenerative disorders, including RGCs neurodegeneration such as glaucoma. In animal model of optic neuropathy (traumatic injury and chronic glaucomatous neurodegeneration) it has been shown that inhibition of the protein kinase RNA-like endoplasmic reticulum kinase (PERK)-eukaryotic initiation factor 2 alpha (eIF2α)-CCAAT/enhancer-binding protein homologous protein (CHOP) pathway and activation of the X-box binding protein 1 (XBP-1) pathway play neuroprotective role in RGCs by preventing ER stress-induced protein misfolding, increasing expression of anti-apoptotic Bcl-2 gene and regulating homeostasis of Ca^2+^ ions in ER (Hu, 2016; Yang et al., 2016). Ojino et al. (2015) additionally presented that in DBA/2J mice, ER stress may participate in optic nerve astrocytes activation, overlapping the direct involvement in axonopathy. Targeting the ER stress pathways by gene therapy approach is considered as a novel and promising direction in neuroprotective strategies.

### Adenosine and Adenosine Receptors

Adenosine is derived from ATP and AMP in the cells and can be transported extracellularly by the nucleoside transporters. It plays role in the regulation of the blood flow, inflammatory cytokine release by T-cells, synaptic function, neuromediators release and Ca^2+^ levels in CNS, as well as in neuroprotection (Sheth et al., 2014). Adenosine receptors (AR) represent class of four known G protein-coupled receptors distributed within different tissues including ocular structures. AR_1_ and AR_3_ receptors stimulation inhibits adenylyl cyclase and decrease cyclic adenosine monophosphate (cAMP) synthesis, while AR_2A_ and AR_2B_ receptors agonists cause activation of adenylyl cyclase and increase cAMP in the cells (Fredholm et al., 2011; Sheth et al., 2014). In the aspect of glaucoma, stimulation of certain subtypes of AR (i.e., agonists of AR_1_ and AR_3,_ and antagonists of AR_2A_) are expected to have beneficial effects on intraocular pressure reduction or directly on RGC neuroprotection (Zhong et al., 2013; Nakashima et al., 2018). The neuroprotective aspects of adenosine are related mostly to AR_1_ and AR_3_ receptors. The molecular neuroprotective effects of AR_1_/AR_3_ agonists are explained by anti-apoptotic effect involving deregulation of PKB/NF-κB and Wnt signaling pathways, downregulation of the Fas receptor, and TNFα expressions (Fishman et al., 2013). Additionally, it has been demonstrated that adenosine signaling (AR_1_ and AR_3_) alleviates excitotoxic insult outcomes by decreasing Ca^2+^ influx, mitochondrial damage and apoptosis related to NMDA or P2X7 receptor stimulation and glutamate accumulation (Hartwick et al., 2004; Zhang et al., 2006, 2010). In the study conducted on *in vitro* RGC culture and rat optic nerve crush model, stimulation of AR_3_ promoted RGC neurites outgrowth which proves that agonists of this receptor may exhibit neuroregenerative activity (Nakashima et al., 2018). Interestingly, agonists of AR_2B_ promotes axons elongation in peripheral neurons, what was demonstrated in corneal nerve plexus in diabetic rats (Zhang et al., 2018).

## Concluding Remarks

There are many candidate molecules and pathways identified as potential therapeutic factors for RGCs rescue. It would be a real challenge to judge and justify which of these represent the most potent one, since it is highly probable that revealed effect depends on the pathomechanism of cell damage. Moreover it is possible that combined therapies might be more effective than single factor application, what was observed, for example in neurotrophic factors therapy (Nishi, 1994; Weissmiller and Wu, 2012). It is important to note that majority of described molecules were tested in animal settings therefore anatomical, physiological and functional differences with human retinas may be associated with the discrepancies between the clinical trials conclusions and animal studies outcomes (i.e., memantine trial in glaucoma (WoldeMussie et al., 2002; Weinreb et al., 2018). From among the factors we described in this review, the human antigen R (HuR protein) seems to be the most promising endogenous target for experimental and translational therapy. In glaucoma, it has been shown that there are similar patterns of HuR protein alterations in both rat and human what allows to expect that effects of HuR-targeted therapies may have similar, thus translational outcomes (Smedowski et al., 2018). HuR protein represent the pleiotropic factor that post-translationally regulates majority of described intracellular processes and molecules, especially antioxidant enzymes, growth factors, stress response factors, cell cycle regulating proteins and autophagy. By modulating single factor – HuR protein expression and function, it is possible to affect whole cell stress-response mechanisms. It seems to be possible HuR protein is a key element managing these complex mechanisms that determine whether cells survive and remain fully functional.

## Author Contributions

MP-D and MA wrote the manuscript text, and created tables and figures. SG and JL-K provided a critical review of the manuscript. AS provided the manuscript concept, designed the draft, and wrote the manuscript text.

## Conflict of Interest Statement

The authors declare that the research was conducted in the absence of any commercial or financial relationships that could be construed as a potential conflict of interest.

## References

[B1] Abdelmohsen K. Kuwano Y. Kim H. H. Gorospe M. (2008). Posttranscriptional gene regulation by RNA-binding proteins during oxidative stress: implications for cellular senescence. *Biol. Chem.* 389 243–255. 10.1515/BC.2008.022 18177264 PMC8481862

[B2] Abu-Amero K. K. Azad T. A. Mousa A. Osman E. A. Sultan T. Al-Obeidan S. A. (2014a). Total antioxidant level is correlated with intra-ocular pressure in patients with primary angle closure glaucoma. *BMC Res. Notes* 7:163. 10.1186/1756-0500-7-163 24646376 PMC3995365

[B3] Abu-Amero K. K. Kondkar A. A. Mousa A. Osman E. A. Al-Obeidan S. A. (2014b). Association of Mn-SOD mutation (c.47T > C) with various POAG clinical indices. *Ophthalmic Genet.* 35 85–90. 10.3109/13816810.2013.796390 23647424

[B4] Abu-Amero K. K. Kondkar A. A. Mousa A. Osman E. A. Al-Obeidan S. A. (2013). Decreased total antioxidants in patients with primary open angle glaucoma. *Curr. Eye Res.* 38 959–964. 10.3109/02713683.2013.794246 23651069

[B5] Agarwal R. Agarwal P. (2012). Glaucomatous neurodegeneration: an eye on tumor necrosis factor-alpha. *Indian J. Ophthalmol.* 60 255–261. 10.4103/0301-4738.98700 22824592 PMC3442458

[B6] Airaksinen M. S. Saarma M. (2002). The GDNF family: signalling, biological functions and therapeutic value. *Nat. Rev. Neurosci.* 3 383–394. 10.1038/nrn812 11988777

[B7] Allen S. J. Watson J. J. Shoemark D. K. Barua N. U. Patel N. K. (2013). GDNF, NGF and BDNF as therapeutic options for neurodegeneration. *Pharmacol. Ther.* 138 155–175. 10.1016/j.pharmthera.2013.01.004 23348013

[B8] Aloe L. Rocco M. Balzamino B. Micera A. (2015). Nerve growth factor: a focus on neuroscience and therapy. *Curr. Neuropharmacol.* 13 294–303. 10.2174/1570159X13666150403231920 26411962 PMC4812798

[B9] Alvarado J. Murphy C. Juster R. (1984). Trabecular meshwork cellularity in primary open-angle glaucoma and nonglaucomatous normals. *Ophthalmology* 91 564–579. 10.1016/S0161-6420(84)34248-8 6462622

[B10] Amadio M. Bucolo C. Leggio G. M. Drago F. Govoni S. Pascale A. (2010). The PKCbeta/HuR/VEGF pathway in diabetic retinopathy. *Biochem. Pharmacol.* 80 1230–1237. 10.1016/j.bcp.2010.06.033 20599775

[B11] Amadio M. Govoni S. Pascale A. (2016a). Targeting VEGF in eye neovascularization: what’s new?: a comprehensive review on current therapies and oligonucleotide-based interventions under development. *Pharmacol. Res.* 103 253–269. 10.1016/j.phrs.2015.11.027 26678602

[B12] Amadio M. Pascale A. Cupri S. Pignatello R. Osera C. D’Agata V. (2016b). Nanosystems based on siRNA silencing HuR expression counteract diabetic retinopathy in rat. *Pharmacol. Res.* 111 713–720. 10.1016/j.phrs.2016.07.042 27475885

[B13] Amadio M. Osera C. Lupo G. Motta C. Drago F. Govoni S. (2012). Protein kinase C activation affects, via the mRNA-binding Hu-antigen R/ELAV protein, vascular endothelial growth factor expression in a pericytic/endothelial coculture model. *Mol. Vis.* 18 2153–2164. 22879735 PMC3415319

[B14] Amadio M. Scapagnini G. Laforenza U. Intrieri M. Romeo L. Govoni S. (2008). Post-transcriptional regulation of HSP70 expression following oxidative stress in SH-SY5Y cells: the potential involvement of the RNA-binding protein HuR. *Curr. Pharm. Des.* 14 2651–2658. 10.2174/138161208786264052 18991684

[B15] Arundine M. Tymianski M. (2003). Molecular mechanisms of calcium-dependent neurodegeneration in excitotoxicity. *Cell Calcium* 34 325–337. 10.1016/S0143-4160(03)00141-6 12909079

[B16] Aydemir O. Nazıroğlu M. Celebi S. Yılmaz T. Kükner A. Ş. (2004). Antioxidant effects of alpha-, gamma- and succinate-tocopherols in guinea pig retina during ischemia-reperfusion injury. *Pathophysiology* 11 167–171. 10.1016/j.pathophys.2004.08.001 15561514

[B17] Bagnis A. Izzotti A. Centofanti M. Saccà S. C. (2012). Aqueous humor oxidative stress proteomic levels in primary open angle glaucoma. *Exp. Eye Res.* 103 55–62. 10.1016/j.exer.2012.07.011 22974818

[B18] Bai Y. Xu J. Brahimi F. Zhuo Y. Sarunic M. V. Uri Saragovi H. (2010). An agonistic TrKb mAb causes sustained TrkB activation, delays RGC death, and protects the retinal structure in optic nerve axotomy and in glaucoma. *Invest. Ophthalmol. Vis. Sci.* 51 4722–4731. 10.1167/iovs.09-5032 20357199

[B19] Bakalash S. Kipnis J. Yoles E. Schwartz M. (2002). Resistance of retinal ganglion cells to an increase in intraocular pressure is immune-dependent. *Invest. Ophthalmol. Vis. Sci.* 43 2648–2653. 12147598

[B20] Bandello F. Lattanzio R. Zucchiatti I. Del Turco C. (2013). Pathophysiology and treatment of diabetic retinopathy. *Acta Diabetol.* 50 1–20. 10.1007/s00592-012-0449-3 23277338

[B21] Barar J. Aghanejad A. Fathi M. Omidi Y. (2016). Advanced drug delivery and targeting technologies for the ocular diseases. *Bioimpacts* 6 49–67. 10.15171/bi.2016.07 27340624 PMC4916551

[B22] Bastaki M. Huen K. Manzanillo P. Chande N. Chen C. Balmes J. R. (2006). Genotype-activity relationship for Mn-superoxide dismutase, glutathione peroxidase 1 and catalase in humans. *Pharmacogenet. Genomics* 16 279–286. 10.1097/01.fpc.0000199498.08725.9c 16538174

[B23] Batcha A. H. Greferath U. Jobling A. I. Vessey K. A. Ward M. M. Nithianantharajah J. (2012). Retinal dysfunction, photoreceptor protein dysregulation and neuronal remodelling in the R6/1 mouse model of Huntington’s disease. *Neurobiol. Dis.* 45 887–896. 10.1016/j.nbd.2011.12.004 22198376

[B24] Batliwala S. Xavier C. Liu Y. Wu H. Pang I. H. (2017). Involvement of Nrf2 in ocular diseases. *Oxid. Med. Cell. Longev.* 2017:1703810. 10.1155/2017/1703810 28473877 PMC5394909

[B25] Beazley-Long N. Hua J. Jehle T. Hulse R. P. Dersch R. Lehrling C. (2013). VEGF-A165b is an endogenous neuroprotective splice isoform of vascular endothelial growth factor a in vivo and in vitro. *Am. J. Pathol.* 183 918–929. 10.1016/j.ajpath.2013.05.031 23838428 PMC3763768

[B26] Becerra S. P. Amaral J. (2002). Erythropoietin–an endogenous retinal survival factor. *N. Engl. J. Med.* 347 1968–1970. 10.1056/NEJMcibr022629 12477950

[B27] Behndig A. Svensson B. Marklund S. L. Karlsson K. (1998). Superoxide dismutase isoenzymes in the human eye. *Invest. Ophthalmol. Vis. Sci.* 39 471–475. 9501855

[B28] Beltran W. A. Zhang Q. Kijas J. W. Gu D. Rohrer H. Jordan J. A. (2003). Cloning, mapping, and retinal expression of the canine ciliary neurotrophic factor receptor alpha (CNTFRalpha). *Invest. Ophthalmol. Vis. Sci.* 44 3642–3649. 10.1167/iovs.02-0763 12882818

[B29] Benoist d’Azy C. Pereira B. Chiambaretta F. Dutheil F. (2016). Oxidative and anti-oxidative stress markers in chronic glaucoma: a systematic review and meta-analysis. *PLoS One* 11:e0166915. 10.1371/journal.pone.0166915 27907028 PMC5131953

[B30] Berk B.-A. Vogler S. Pannicke T. Kuhrt H. Garcia T. B. Wiedemann P. (2015). Brain-derived neurotrophic factor inhibits osmotic swelling of rat retinal glial (Müller) and bipolar cells by activation of basic fibroblast growth factor signaling. *Neuroscience* 295 175–186. 10.1016/j.neuroscience.2015.03.037 25813711

[B31] Bernstein S. L. Mehrabyan Z. Guo Y. Moianie N. (2007). Estrogen is not neuroprotective in a rodent model of optic nerve stroke. *Mol. Vis.* 13 1920–1925. 17982415 PMC2185481

[B32] Birch D. G. Bennett L. D. Duncan J. L. Weleber R. G. Pennesi M. E. (2016). Long-term follow-up of patients with retinitis pigmentosa receiving intraocular ciliary neurotrophic factor implants. *Am. J. Ophthalmol.* 170 10–14. 10.1016/j.ajo.2016.07.013 27457255 PMC5056139

[B33] Birch D. G. Weleber R. G. Duncan J. L. Jaffe G. J. Tao W. (2013). Randomized trial of ciliary neurotrophic factor delivered by encapsulated cell intraocular implants for retinitis pigmentosa. *Am. J. Ophthalmol.* 156 283–292.e1. 10.1016/j.ajo.2013.03.021 23668681 PMC4111936

[B34] Bouayed J. Bohn T. (2010). Exogenous antioxidants—double-edged swords in cellular redox state: health beneficial effects at physiologic doses versus deleterious effects at high doses. *Oxid. Med. Cell. Longev.* 3 228–237. 10.4161/oxim.3.4.12858 20972369 PMC2952083

[B35] Brar V. S. Sharma R. K. Murthy R. K. Chalam K. V. (2010). Bevacizumab neutralizes the protective effect of vascular endothelial growth factor on retinal ganglion cells. *Mol. Vis.* 16 1848–1853. 21031022 PMC2956671

[B36] Bruno M. A. Cuello A. C. (2006). Activity-dependent release of precursor nerve growth factor, conversion to mature nerve growth factor, and its degradation by a protease cascade. *Proc. Natl. Acad. Sci. U.S.A.* 103 6735–6740. 10.1073/pnas.0510645103 16618925 PMC1458950

[B37] Carelli V. Ross-Cisneros F. N. Sadun A. A. (2004). Mitochondrial dysfunction as a cause of optic neuropathies. *Prog. Retin. Eye Res.* 23 53–89. 10.1016/j.preteyeres.2003.10.003 14766317

[B38] Cécyre B. Zabouri N. Huppé-Gourgues F. Bouchard J.-F. Casanova C. (2013). Roles of cannabinoid receptors type 1 and 2 on the retinal function of adult mice. *Invest. Ophthalmol. Vis. Sci.* 54 8079–8090. 10.1167/iovs.13-12514 24255040

[B39] Chan H. C. Chang R. C. C. Koon-Ching Ip A. Chiu K. Yuen W. H. Zee S. Y. (2007). Neuroprotective effects of *Lycium barbarum* Lynn on protecting retinal ganglion cells in an ocular hypertension model of glaucoma. *Exp. Neurol.* 203 269–273. 10.1016/j.expneurol.2006.05.031 17045262

[B40] Chang E. E. Goldberg J. L. (2012). Glaucoma 2.0: neuroprotection, neuroregeneration, neuroenhancement. *Ophthalmology* 119 979–986. 10.1016/j.ophtha.2011.11.003 22349567 PMC3343191

[B41] Chang Z. Y. Yeh M. K. Chiang C. H. Chen Y. H. Lu D. W. (2013). Erythropoietin protects adult retinal ganglion cells against NMDA-, trophic factor withdrawal-, and TNF-α-induced damage. *PLoS One* 8:e55291. 10.1371/journal.pone.0055291 23383140 PMC3559395

[B42] Chen L. Qi Y. Yang X. (2015). Neuroprotective effects of crocin against oxidative stress induced by ischemia/reperfusion injury in rat retina. *Ophthalmic Res.* 54 157–168. 10.1159/000439026 26437379

[B43] Chen Q. Wang H. Liao S. Gao Y. Liao R. Little P. J. (2015). Nerve growth factor protects retinal ganglion cells against injury induced by retinal ischemia-reperfusion in rats. *Growth Factors* 33 149–159. 10.3109/08977194.2015.1010642 25707536

[B44] Chew E. Y. Clemons T. E. Peto T. Sallo F. B. Ingerman A. Tao W. (2015). Ciliary neurotrophic factor for macular telangiectasia type 2: results from a phase 1 safety trial. *Am. J. Ophthalmol.* 159 659–666. 10.1016/j.ajo.2014.12.013 25528956 PMC4361328

[B45] Chidlow G. Wood J. P. M. Casson R. J. (2014). Expression of inducible heat shock proteins Hsp27 and Hsp70 in the visual pathway of rats subjected to various models of retinal ganglion cell injury. *PLoS One* 9:e114838. 10.1371/journal.pone.0114838 25535743 PMC4275305

[B46] Cho H. Hartsock M. J. Xu Z. He M. Duh E. J. (2015). Monomethyl fumarate promotes Nrf2-dependent neuroprotection in retinal ischemia-reperfusion. *J. Neuroinflammation* 12:239. 10.1186/s12974-015-0452-z 26689280 PMC4687295

[B47] Chou T. H. Musada G. R. Romano G. L. Bolton E. Porciatti V. (2018). Anesthetic preconditioning as endogenous neuroprotection in glaucoma. *Int. J. Mol. Sci.* 19:E237. 10.3390/ijms19010237 29342845 PMC5796185

[B48] Coassin M. Lambiase A. Sposato V. Micera A. Bonini S. Aloe L. (2008). Retinal p75 and bax overexpression is associated with retinal ganglion cells apoptosis in a rat model of glaucoma. *Graefes Arch. Clin. Exp. Ophthalmol.* 246 1743–1749. 10.1007/s00417-008-0913-5 18751719

[B49] Colafrancesco V. Parisi V. Sposato V. Rossi S. Russo M. A. Coassin M. (2011). Ocular application of nerve growth factor protects degenerating retinal ganglion cells in a rat model of glaucoma. *J. Glaucoma* 20 100–108. 10.1097/IJG.0b013e3181d787e5 20436364

[B50] Crish S. D. Calkins D. J. (2011). Neurodegeneration in glaucoma: progression and calcium-dependent intracellular mechanisms. *Neuroscience* 176 1–11. 10.1016/j.neuroscience.2010.12.036 21187126 PMC3040267

[B51] Cueva Vargas J. L. Osswald I. K. Unsain N. Aurousseau M. R. Barker P. A. Bowie D. (2015). Soluble tumor necrosis factor alpha promotes retinal ganglion cell death in glaucoma via calcium-permeable AMPA receptor activation. *J. Neurosci.* 35 12088–12102. 10.1523/JNEUROSCI.1273-15.2015 26338321 PMC6605307

[B52] Cwerman-Thibault H. Lechauve C. Augustin S. Roussel D. Reboussin É. Mohammad A. (2017). Neuroglobin can prevent or reverse glaucomatous progression in DBA/2J mice. *Mol. Ther. Methods Clin. Dev.* 5 200–220. 10.1016/j.omtm.2017.04.008 28540323 PMC5430497

[B53] Dai M. Xia X. B. Xiong S. Q. (2012). BDNF regulates GLAST and glutamine synthetase in mouse retinal Müller cells. *J. Cell. Physiol.* 227 596–603. 10.1002/jcp.22762 21448920

[B54] Daugherty C. L. Curtis H. Realini T. Charlton J. F. Zareparsi S. (2009). Primary open angle glaucoma in a Caucasian population is associated with the p53 codon 72 polymorphism. *Mol. Vis.* 15 1939–1944. 19784392 PMC2751801

[B55] Davis B. M. Crawley L. Pahlitzsch M. Javaid F. Cordeiro M. F. (2016). Glaucoma: the retina and beyond. *Acta Neuropathol.* 132 807–826. 10.1007/s00401-016-1609-2 27544758 PMC5106492

[B56] De Conti L. Baralle M. Buratti E. (2017). Neurodegeneration and RNA-binding proteins. *Wiley Interdiscip. Rev. RNA* 8:e1394. 10.1002/wrna.1394 27659427

[B57] de Voogd S. Wolfs R. C. W. Jansonius N. M. Uitterlinden A. G. Pols H. A. P. Hofman A. (2008). Estrogen receptors alpha and beta and the risk of open-angle glaucoma: the Rotterdam Study. *Arch. Ophthalmol.* 126 110–114. 10.1001/archopht.126.1.110 18195227

[B58] Deng S. Wang M. Yan Z. Tian Z. Chen H. Yang X. (2013). Autophagy in retinal ganglion cells in a rhesus monkey chronic hypertensive glaucoma model. *PLoS One* 8:e77100. 10.1371/journal.pone.0077100 24143204 PMC3797129

[B59] Dilsiz N. Sahaboglu A. Yildiz M. Z. Reichenbach A. (2006). Protective effects of various antioxidants during ischemia-reperfusion in the rat retina. *Graefes Arch. Clin. Exp. Ophthalmol.* 244 627–633. 10.1007/s00417-005-0084-6 16205934

[B60] Doller A. Pfeilschifter J. Eberhardt W. (2008). Signalling pathways regulating nucleo-cytoplasmic shuttling of the mRNA-binding protein HuR. *Cell. Signal.* 20 2165–2173. 10.1016/j.cellsig.2008.05.007 18585896

[B61] Domenici L. Origlia N. Falsini B. Cerri E. Barloscio D. Fabiani C. (2014). Rescue of retinal function by BDNF in a mouse model of glaucoma. *PLoS One* 9:e115579. 10.1371/journal.pone.0115579 25536045 PMC4275209

[B62] Dong C. J. Guo Y. Agey P. Wheeler L. Hare W. A. (2008). α2 adrenergic modulation of NMDA receptor function as a major mechanism of RGC protection in experimental glaucoma and retinal excitotoxicity. *Invest. Ophthalmol. Vis. Sci.* 49 4515–4522. 10.1167/iovs.08-2078 18566471

[B63] El-Hattab A. W. Zarante A. M. Almannai M. Scaglia F. (2017). Therapies for mitochondrial diseases and current clinical trials. *Mol. Genet. Metab.* 122 1–9. 10.1016/j.ymgme.2017.09.009 28943110 PMC5773113

[B64] El-Remessy A. B. Al-Shabrawey M. Khalifa Y. Tsai N. T. Caldwell R. B. Liou G. I. (2006). Neuroprotective and blood-retinal barrier-preserving effects of cannabidiol in experimental diabetes. *Am. J. Pathol.* 168 235–244. 10.2353/ajpath.2006.050500 16400026 PMC1592672

[B65] Erdurmuş M. Yağcı R. Atış Ö. Karadağ R. Akbaş A. Hepşen İ. F. (2011). Antioxidant status and oxidative stress in primary open angle glaucoma and pseudoexfoliative glaucoma. *Curr. Eye Res.* 36 713–718. 10.3109/02713683.2011.584370 21780920

[B66] Erslev A. J. Caro J. (1986). Physiologic and molecular biology of erythropoietin. *Med. Oncol. Tumor Pharmacother.* 3 159–164. 10.1007/BF02934992 3543529

[B67] Fahnestock M. Michalski B. Xu B. Coughlin M. D. (2001). The precursor pro-nerve growth factor is the predominant form of nerve growth factor in brain and is increased in Alzheimer’s disease. *Mol. Cell. Neurosci.* 18 210–220. 10.1006/mcne.2001.1016 11520181

[B68] Famiglietti E. V. Stopa E. G. McGookin E. D. Song P. LeBlanc V. Streeten B. W. (2003). Immunocytochemical localization of vascular endothelial growth factor in neurons and glial cells of human retina. *Brain Res.* 969 195–204. 10.1016/S0006-8993(02)03766-6 12676380

[B69] Fan B. J. Liu K. Wang D. Y. Tham C. C. Y. Tam P. O. S. Lam D. S. C. (2010). Association of polymorphisms of tumor necrosis factor and tumor protein p53 with primary open-angle glaucoma. *Invest. Ophthalmol. Vis. Sci.* 51 4110–4116. 10.1167/iovs.09-4974 20357201

[B70] Ferrara N. Gerber H. P. LeCouter J. (2003). The biology of VEGF and its receptors. *Nat. Med.* 9 669–676. 10.1038/nm0603-669 12778165

[B71] Ferreira S. M. Lerner F. Reides C. G. Brunzini R. Llesuy S. F. (2013). Brain antioxidant status in a high pressure-induced rat model of glaucoma. *Acta Ophthalmol.* 91 e64–e70. 10.1111/j.1755-3768.2012.02572.x 23025455

[B72] Ferreira S. M. Lerner S. F. Brunzini R. Evelson P. A. Llesuy S. F. (2004). Oxidative stress markers in aqueous humor of glaucoma patients. *Am. J. Ophthalmol.* 137 62–69. 10.1016/S0002-9394(03)00788-8 14700645

[B73] Fishman P. Cohen S. Bar-Yehuda S. (2013). Targeting the A3 adenosine receptor for glaucoma treatment (Review). *Mol. Med. Rep.* 7 1723–1725. 10.3892/mmr.2013.1413 23563604

[B74] Fitzgerald M. Nairn P. Bartlett C. A. Chung R. S. West A. K. Beazley L. D. (2007). Metallothionein-IIA promotes neurite growth via the megalin receptor. *Exp. Brain Res.* 183 171–180. 10.1007/s00221-007-1032-y 17634932

[B75] Fogli S. Del Re M. Rofi E. Posarelli C. Figus M. Danesi R. (2018). Clinical pharmacology of intravitreal anti-VEGF drugs. *Eye* 32 1010–1020. 10.1038/s41433-018-0021-7 29398697 PMC5997665

[B76] Fontaine S. N. Martin M. D. Dickey C. A. (2016). Neurodegeneration and the heat shock protein 70 machinery: implications for therapeutic development. *Curr. Top. Med. Chem.* 16 2741–2752. 10.2174/1568026616666160413140741 27072702

[B77] Foxton R. H. Finkelstein A. Vijay S. Dahlmann-Noor A. Khaw P. T. Morgan J. E. (2013). VEGF-A is necessary and sufficient for retinal neuroprotection in models of experimental glaucoma. *Am. J. Pathol.* 182 1379–1390. 10.1016/j.ajpath.2012.12.032 23416159 PMC3608027

[B78] Fredholm B. B. IJzerman A. P. Jacobson K. A. Linden J. Muller C. E. (2011). International union of basic and clinical pharmacology. LXXXI. Nomenclature and classification of adenosine receptors–an update. *Pharmacol. Rev.* 63 1–34. 10.1124/pr.110.003285 21303899 PMC3061413

[B79] Freeman R. S. Burch R. L. Crowder R. J. Lomb D. J. Schoell M. C. Straub J. A. (2004). NGF deprivation-induced gene expression: after ten years, where do we stand? *Prog. Brain Res.* 146 111–126. 10.1016/S0079-6123(03)46008-1 14699960

[B80] Froger N. Cadetti L. Lorach H. Martins J. Bemelmans A. P. Dubus E. (2012). Taurine provides neuroprotection against retinal ganglion cell degeneration. *PLoS One* 7:e42017. 10.1371/journal.pone.0042017 23115615 PMC3480351

[B81] Froger N. Jammoul F. Gaucher D. Cadetti L. Lorach H. Degardin J. (2013). Taurine is a crucial factor to preserve retinal ganglion cell survival. *Adv. Exp. Med. Biol.* 775 69–83. 10.1007/978-1-4614-6130-2_6 23392925

[B82] Fu Q. L. Wu W. Wang H. Li X. Lee V. W. So K. F. (2008). Up-regulated endogenous erythropoietin/erythropoietin receptor system and exogenous erythropoietin rescue retinal ganglion cells after chronic ocular hypertension. *Cell. Mol. Neurobiol.* 28 317–329. 10.1007/s10571-007-9155-z 17554621 PMC11515015

[B83] Fujita K. Nishiguchi K. M. Shiga Y. Nakazawa T. (2017). Spatially and temporally regulated NRF2 gene therapy using Mcp-1 promoter in retinal ganglion cell injury. *Mol. Ther. Methods Clin. Dev.* 5 130–141. 10.1016/j.omtm.2017.04.003 28480312 PMC5415330

[B84] Garcia T. B. Pannicke T. Vogler S. Berk B. A. Grosche A. Wiedemann P. (2014). Nerve growth factor inhibits osmotic swelling of rat retinal glial (Müller) and bipolar cells by inducing glial cytokine release. *J. Neurochem.* 131 303–313. 10.1111/jnc.12822 25041175

[B85] Gauthier A. C. Liu J. (2017). Epigenetics and signaling pathways in glaucoma. *Biomed Res. Int.* 2017:5712341. 10.1155/2017/5712341 28210622 PMC5292191

[B86] Gemenetzi M. Lotery A. J. Patel P. J. (2017). Risk of geographic atrophy in age-related macular degeneration patients treated with intravitreal anti-VEGF agents. *Eye* 31 1–9. 10.1038/eye.2016.208 27716750 PMC5233933

[B87] Gerber A. L. Harris A. Siesky B. Lee E. Schaab T. J. Huck A. (2015). Vascular dysfunction in diabetes and glaucoma: a complex relationship reviewed. *J. Glaucoma* 24 474–479. 10.1097/IJG.0000000000000137 25264988

[B88] Ghaffariyeh A. Honarpisheh N. Heidari M. H. Puyan S. Abasov F. (2011). Brain-derived neurotrophic factor as a biomarker in primary open-angle glaucoma. *Optom. Vis. Sci.* 88 80–85. 10.1097/OPX.0b013e3181fc329f 21076359

[B89] Ghaffariyeh A. Honarpisheh N. Shakiba Y. Puyan S. Chamacham T. Zahedi F. (2009). Brain-derived neurotrophic factor in patients with normal-tension glaucoma. *Optometry* 80 635–638. 10.1016/j.optm.2008.09.014 19861219

[B90] Ghanem A. A. Arafa L. F. El-Baz A. (2010). Oxidative stress markers in patients with primary open-angle glaucoma. *Curr. Eye Res.* 35 295–301. 10.3109/02713680903548970 20373896

[B91] Gherghel D. Griffiths H. R. Hilton E. J. Cunliffe I. A. Hosking S. L. (2005). Systemic reduction in glutathione levels occurs in patients with primary open-angle glaucoma. *Invest. Ophthalmol. Vis. Sci.* 46 877–883. 10.1167/iovs.04-0777 15728543

[B92] Gionfriddo J. R. Freeman K. S. Groth A. Scofield V. L. Alyahya K. Madl J. E. (2009). α-Luminol prevents decreases in glutamate, glutathione, and glutamine synthetase in the retinas of glaucomatous DBA/2J mice. *Vet. Ophthalmol.* 12 325–332. 10.1111/j.1463-5224.2009.00722.x 19751494

[B93] Gohari M. Neamatzadeh H. Ali Jafari M. Mazaheri M. Zare-Shehneh M. Abbasi-Shavazi E. (2016). Association between the p53 codon 72 polymorphism and primary open-angle glaucoma risk: meta-analysis based on 11 case–control studies. *Indian J. Ophthalmol.* 64 756–761. 10.4103/0301-4738.195002 27905339 PMC5168918

[B94] Gómez del Pulgar T. Velasco G. Guzmán M. (2000). The CB1 cannabinoid receptor is coupled to the activation of protein kinase B/Akt. *Biochem. J.* 347 369–373. 10.1016/S0960-9822(00)00499-1 10749665 PMC1220968

[B95] Goyal A. Srivastava A. Sihota R. Kaur J. (2014). Evaluation of oxidative stress markers in aqueous humor of primary open angle glaucoma and primary angle closure glaucoma patients. *Curr. Eye Res.* 39 823–829. 10.3109/02713683.2011.556299 24912005

[B96] Greenberg M. E. Xu B. Lu B. Hempstead B. L. (2009). New insights in the biology of BDNF synthesis and release: implications in CNS function. *J. Neurosci.* 29 12764–12767. 10.1523/JNEUROSCI.3566-09.2009 19828787 PMC3091387

[B97] Guo X. Kimura A. Azuchi Y. Akiyama G. Noro T. Harada C. (2016). Caloric restriction promotes cell survival in a mouse model of normal tension glaucoma. *Sci. Rep.* 6:33950. 10.1038/srep33950 27669894 PMC5037377

[B98] Guo X. Kong X. Huang R. Jin L. Ding X. He M. (2014). Effect of *Ginkgo biloba* on visual field and contrast sensitivity in Chinese patients with normal tension glaucoma: a randomized, crossover clinical trial. *Invest. Ophthalmol. Vis. Sci.* 55 110–116. 10.1167/iovs.13-13168 24282229

[B99] Guo X. J. Tian X. S. Ruan Z. Chen Y. T. Wu L. Gong Q. (2014). Dysregulation of neurotrophic and inflammatory systems accompanied by decreased CREB signaling in ischemic rat retina. *Exp. Eye Res.* 125 156–63 10.1016/j.exer.2014.06.003 24954538

[B100] Guo Y. Zhang H. Chen X. Yang X. Cheng W. Zhao K. (2012). Association of TP53 polymorphisms with primary open-angle glaucoma: a meta-analysis. *Invest. Ophthalmol. Vis. Sci.* 53 3756–3763. 10.1167/iovs.12-9818 22562509

[B101] Gupta P. D. Johar K. Nagpal K. Vasavada A. R. (2005). Sex hormone receptors in the human eye. *Surv. Ophthalmol.* 50 274–284. 10.1016/j.survophthal.2005.02.005 15850816

[B102] Gupta V. Gupta V. B. Chitranshi N. Gangoda S. Vander Wall R. Abbasi M. (2016). One protein, multiple pathologies: multifaceted involvement of amyloid β in neurodegenerative disorders of the brain and retina. *Cell. Mol. Life Sci.* 73 4279–4297. 10.1007/s00018-016-2295-x 27333888 PMC11108534

[B103] Gupta V. You Y. Li J. Gupta V. Golzan M. Klistorner A. (2014). BDNF impairment is associated with age-related changes in the inner retina and exacerbates experimental glaucoma. *Biochim. Biophys. Acta Mol. Basis Dis.* 1842 1567–1578. 10.1016/j.bbadis.2014.05.026 24942931

[B104] Halliwell B. Gutteridge J. M. (1999). Free radicals in biology and medicine. *Free Radic. Biol. Med.* 10 449–450. 10.1016/0748-5514(85)90028-5 3939136

[B105] Hao M. Li Y. Lin W. Xu Q. Shao N. Zhang Y. (2014). Estrogen prevents high-glucose-induced damage of retinal ganglion cells via mitochondrial pathway. *Graefes Arch. Clin. Exp. Ophthalmol.* 253 83–90. 10.1007/s00417-014-2771-7 25216739

[B106] Hart N. J. Koronyo Y. Black K. L. Koronyo-Hamaoui M. (2016). Ocular indicators of Alzheimer’s: exploring disease in the retina. *Acta Neuropathol.* 132 767–787. 10.1007/s00401-016-1613-6 27645291 PMC5106496

[B107] Hartwick A. T. E. Lalonde M. R. Barnes S. Baldridge W. H. (2004). Adenosine A1-receptor modulation of glutamate-induced calcium influx in rat retinal ganglion cells. *Invest. Ophthalmol. Vis. Sci.* 45 3740–3748. 10.1167/iovs.04-0214 15452085

[B108] Harvey S. Parker E. Macdonald I. Sanders E. J. (2009). Growth hormone is present in the human retina and vitreous fluid. *Neurosci. Lett.* 455 199–202. 10.1016/j.neulet.2009.03.073 19429121

[B109] Hayashi H. Takagi N. (2015). Endogenous neuroprotective molecules and their mechanisms in the central nervous system. *Biol. Pharm. Bull.* 38 1104–1108. 10.1248/bpb.b15-00361 26235573

[B110] Helal G. K. Aleisa A. M. Helal O. K. Al-Rejaie S. S. Al-Yahya A. A. Al-Majed A. A. (2009). Metallothionein induction reduces caspase-3 activity and TNFalpha levels with preservation of cognitive function and intact hippocampal neurons in carmustine-treated rats. *Oxid. Med. Cell. Longev.* 2 26–35. 10.4161/oxim.2.1.7901 20046642 PMC2763228

[B111] Herzog K. H. von Bartheld C. S. (1998). Contributions of the optic tectum and the retina as sources of brain-derived neurotrophic factor for retinal ganglion cells in the chick embryo. *J. Neurosci.* 18 2891–2906. 10.1523/JNEUROSCI.18-08-02891.1998 9526006 PMC6792593

[B112] Himori N. Yamamoto K. Maruyama K. Ryu M. Taguchi K. Yamamoto M. (2013). Critical role of Nrf2 in oxidative stress-induced retinal ganglion cell death. *J. Neurochem.* 127 669–680. 10.1111/jnc.12325 23721546

[B113] Hines-Beard J. Bond W. S. Backstrom J. R. Rex T. S. (2016). Virus-mediated EpoR76E gene therapy preserves vision in a glaucoma model by modulating neuroinflammation and decreasing oxidative stress. *J. Neuroinflammation* 13:39. 10.1186/s12974-016-0499-5 26876380 PMC4753658

[B114] Hirooka K. Tokuda M. Miyamoto O. Itano T. Baba T. Shiraga F. (2004). The *Ginkgo biloba* extract (EGb 761) provides a neuroprotective effect on retinal ganglion cells in a rat model of chronic glaucoma. *Curr. Eye Res.* 28 153–157. 10.1076/ceyr.28.3.153.26246 14977516

[B115] Hu Y. (2016). Axon injury induced endoplasmic reticulum stress and neurodegeneration. *Neural Regen. Res.* 11 1557–1559. 10.4103/1673-5374.193225 27904477 PMC5116825

[B116] Ikwegbue P. Masamba P. Oyinloye B. Kappo A. (2017). Roles of heat shock proteins in apoptosis, oxidative stress, human inflammatory diseases, and cancer. *Pharmaceuticals* 11:E2. 10.3390/ph11010002 29295496 PMC5874698

[B117] Inman D. M. Lambert W. S. Calkins D. J. Horner P. J. (2013). α-lipoic acid antioxidant treatment limits glaucoma-related retinal ganglion cell death and dysfunction. *PLoS One* 8:e65389. 10.1371/journal.pone.0065389 23755225 PMC3673940

[B118] Izzotti A. Saccà S. C. Di Marco B. Penco S. Bassi A. M. (2008). Antioxidant activity of timolol on endothelial cells and its relevance for glaucoma course. *Eye* 22 445–453. 10.1038/sj.eye.6702737 17293786

[B119] Jacquier-Sarlin M. R. Fuller K. Dinh-Xuan A. T. Richard M. J. Polla B. S. (1994). Protective effects of hsp70 in inflammation. *Experientia* 50 1031–1038. 10.1007/BF01923458 7988662

[B120] Jayaram H. Phillips J. I. Lozano D. C. Choe T. E. Cepurna W. O. Johnson E. C. (2017). Comparison of MicroRNA expression in aqueous humor of normal and primary open-angle glaucoma patients using PCR arrays: a pilot study. *Invest. Ophthalmol. Vis. Sci.* 58 2884–2890. 10.1167/iovs.17-21844 28586912 PMC5460954

[B121] Ji J.-Z. Elyaman W. Yip H. K. Lee V. W. Yick L.-W. Hugon J. (2004). CNTF promotes survival of retinal ganglion cells after induction of ocular hypertension in rats: the possible involvement of STAT3 pathway. *Eur. J. Neurosci.* 19 265–272. 10.1111/j.1460-9568.2003.03107.x 14725620

[B122] Jiang W. Tang L. Zeng J. Chen B. (2016). Adeno-associated virus mediated SOD gene therapy protects the retinal ganglion cells from chronic intraocular pressure elevation induced injury via attenuating oxidative stress and improving mitochondrial dysfunction in a rat model. *Am. J. Transl. Res.* 8 799–810. 27158370 PMC4846927

[B123] Jindal N. Banik A. Prabhakar S. Vaiphie K. Anand A. (2017). Alteration of neurotrophic factors after transplantation of bone marrow derived lin-ve stem cell in nmda-induced mouse model of retinal degeneration. *J. Cell. Biochem.* 118 1699–1711. 10.1002/jcb.25827 27935095

[B124] Jo A. O. Noel J. M. Lakk M. Yarishkin O. Ryskamp D. A. Shibasaki K. (2017). Mouse retinal ganglion cell signalling is dynamically modulated through parallel anterograde activation of cannabinoid and vanilloid pathways. *J. Physiol.* 595 6499–6516. 10.1113/JP274562 28766743 PMC5638913

[B125] Johnson E. C. Deppmeier L. M. Wentzien S. K. Hsu I. Morrison J. C. (2000). Chronology of optic nerve head and retinal responses to elevated intraocular pressure. *Invest. Ophthalmol. Vis. Sci.* 41 431–442. Available at: http://www.ncbi.nlm.nih.gov/pubmed/10670473 10670473

[B126] Johnson T. V. Bull N. D. Martin K. R. (2011). Neurotrophic factor delivery as a protective treatment for glaucoma. *Exp. Eye Res.* 93 196–203. 10.1016/j.exer.2010.05.016 20685205

[B127] Joseph R. Srivastava O. P. Pfister R. R. (2012). Downregulation of β-actin gene and human antigen R in human keratoconus. *Invest. Ophthalmol. Vis. Sci.* 53 4032–4041. 10.1167/iovs.11-9062 22562506 PMC4625805

[B128] Junk A. K. Mammis A. Savitz S. I. Singh M. Roth S. Malhotra S. (2002). Erythropoietin administration protects retinal neurons from acute ischemia-reperfusion injury. *Proc. Natl. Acad. Sci. U.S.A.* 99 10659–10664. 10.1073/pnas.152321399 12130665 PMC125005

[B129] Kaarniranta K. Salminen A. Eskelinen E. L. Kopitz J. (2009). Heat shock proteins as gatekeepers of proteolytic pathways-Implications for age-related macular degeneration (AMD). *Ageing Res. Rev.* 8 128–139. 10.1016/j.arr.2009.01.001 19274853

[B130] Kampinga H. H. Bergink S. (2016). Heat shock proteins as potential targets for protective strategies in neurodegeneration. *Lancet Neurol.* 15 748–759. 10.1016/S1474-4422(16)00099-5 27106072

[B131] Kang J. M. Lin S. (2018). *Ginkgo biloba* and its potential role in glaucoma. *Curr. Opin. Ophthalmol.* 29 116–120. 10.1097/ICU.0000000000000459 29206653

[B132] Karanian D. A. (2005). Dual modulation of endocannabinoid transport and fatty acid amide hydrolase protects against excitotoxicity. *J. Neurosci.* 25 7813–7820. 10.1523/JNEUROSCI.2347-05.2005 16120783 PMC6725251

[B133] Kauper K. McGovern C. Sherman S. Heatherton P. Rapoza R. Stabila P. (2012). Two-year intraocular delivery of ciliary neurotrophic factor by encapsulated cell technology implants in patients with chronic retinal degenerative diseases. *Invest. Ophthalmol. Vis. Sci.* 53 7484–7491. 10.1167/iovs.12-9970 23049090

[B134] Kilic U. Kilic E. Soliz J. Bassetti C. I. Gassmann M. Hermann D. M. (2005). Erythropoietin protects from axotomy-induced degeneration of retinal ganglion cells by activating ERK-1/-2. *FASEB J.* 19 249–251. 10.1096/fj.04-2493fje 15556972

[B135] Kim S. J. Ko J. H. Yun J. H. Kim J. A. Kim T. E. Lee H. J. (2013). Stanniocalcin-1 protects retinal ganglion cells by inhibiting apoptosis and oxidative damage. *PLoS One* 8:e63749. 10.1371/journal.pone.0063749 23667669 PMC3646795

[B136] Kimura A. Namekata K. Guo X. Harada C. Harada T. (2016). Neuroprotection, growth factors and BDNF-TRKB signalling in retinal degeneration. *Int. J. Mol. Sci.* 17:E1584. 10.3390/ijms17091584 27657046 PMC5037849

[B137] Kleesattel D. Crish S. D. Inman D. M. (2015). Decreased energy capacity and increased autophagic activity in optic nerve axons with defective anterograde transport. *Invest. Ophthalmol. Vis. Sci.* 56 8215–8227. 10.1167/iovs.15-17885 26720474 PMC5110237

[B138] Koch J. C. Lingor P. (2016). The role of autophagy in axonal degeneration of the optic nerve. *Exp. Eye Res.* 144 81–89. 10.1016/j.exer.2015.08.016 26315785

[B139] Koeberle P. D. Bähr M. (2008). The upregulation of GLAST-1 is an indirect antiapoptotic mechanism of GDNF and neurturin in the adult CNS. *Cell Death Differ.* 15 471–483. 10.1038/sj.cdd.4402281 18064044

[B140] Kokona D. Georgiou P. C. Kounenidakis M. Kiagiadaki F. Thermos K. (2016). Endogenous and synthetic cannabinoids as therapeutics in retinal disease. *Neural Plast* 2016:8373020. 10.1155/2016/8373020 26881135 PMC4736800

[B141] Kretz A. Kügler S. Happold C. Bähr M. Isenmann S. (2004). Excess Bcl-XL increases the intrinsic growth potential of adult CNS neurons in vitro. *Mol. Cell. Neurosci.* 26 63–74. 10.1016/j.mcn.2004.01.007 15121179

[B142] Krishnan G. Chatterjee N. (2012). Endocannabinoids alleviate proinflammatory conditions by modulating innate immune response in muller glia during inflammation. *Glia* 60 1629–1645. 10.1002/glia.22380 22807196

[B143] Kwong J. M. K. Caprioli J. (2006). Expression of phosphorylated c-Jun N-terminal protein kinase (JNK) in experimental glaucoma in rats. *Exp. Eye Res.* 82 576–582. 10.1016/j.exer.2005.08.017 16197943

[B144] Lebedeva S. Jens M. Theil K. Schwanhäusser B. Selbach M. Landthaler M. (2011). Transcriptome-wide analysis of regulatory interactions of the RNA-binding protein HuR. *Mol. Cell* 43 340–352. 10.1016/j.molcel.2011.06.008 21723171

[B145] Lechauve C. Augustin S. Cwerman-Thibault H. Bouaita A. Forster V. Célier C. (2012). Neuroglobin involvement in respiratory chain function and retinal ganglion cell integrity. *Biochim. Biophys. Acta Mol. Cell Res.* 1823 2261–2273. 10.1016/j.bbamcr.2012.09.009 23036890

[B146] Lee J. Sohn S. W. Kee C. (2012). Effect of *Ginkgo biloba* extract on visual field progression in normal tension glaucoma. *J. Glaucoma* 22 780–784. 10.1097/IJG.0b013e3182595075 22595937

[B147] Lee J. M. Bae H. W. Lee S. Y. Seong G. J. Kim C. Y. (2017). Effect of anti-vascular endothelial growth factor antibody on the survival of cultured retinal ganglion cells. *Korean J. Ophthalmol.* 31 360–365. 10.3341/kjo.2017.0054 28752700 PMC5540992

[B148] Lee W. J. Kim Y. K. Kim Y. W. Jeoung J. W. Kim S. H. Heo J. W. (2017). Rate of macular ganglion cell-inner plexiform layer thinning in glaucomatous eyes with vascular endothelial growth factor inhibition. *J. Glaucoma* 26 980–986. 10.1097/IJG.0000000000000776 28930884

[B149] Levkovitch-Verbin H. (2015). Retinal ganglion cell apoptotic pathway in glaucoma: initiating and downstream mechanisms. *Prog. Brain Res.* 220 37–57. 10.1016/bs.pbr.2015.05.005 26497784

[B150] Levkovitch-Verbin H. Harizman N. Dardik R. Nisgav Y. Vander S. Melamed S. (2007). Regulation of cell death and survival pathways in experimental glaucoma. *Exp. Eye Res.* 85 250–258. 10.1016/j.exer.2007.04.011 17586494

[B151] Levkovitch-Verbin H. Harris-Cerruti C. Groner Y. Wheeler L. A. Schwartz M. Yoles E. (2000). RGC death in mice after optic nerve crush injury: oxidative stress and neuroprotection. *Invest. Ophthalmol. Vis. Sci.* 41 4169–4174. 11095611

[B152] Levkovitch-Verbin H. Vander S. Makarovsky D. Lavinsky F. (2013). Increase in retinal ganglion cells’ susceptibility to elevated intraocular pressure and impairment of their endogenous neuroprotective mechanism by age. *Mol. Vis.* 19 2011–2022. 24146536 PMC3783363

[B153] Li H. Wang B. Zhu C. Feng Y. Wang S. Shahzad M. (2013). 17β-estradiol impedes Bax-involved mitochondrial apoptosis of retinal nerve cells induced by oxidative damage via the phosphatidylinositol 3-kinase/Akt signal pathway. *J. Mol. Neurosci.* 50 482–493. 10.1007/s12031-013-9968-9 23361188

[B154] Li R. Wen R. Banzon T. Maminishkis A. Miller S. S. (2011). CNTF mediates neurotrophic factor secretion and fluid absorption in human retinal pigment epithelium. *PLoS One* 6:e23148. 10.1371/journal.pone.0023148 21912637 PMC3166283

[B155] Libby R. T. Li Y. Savinova O. V. Barter J. Smith R. S. Nickells R. W. (2005). Susceptibility to neurodegeneration in a glaucoma is modified by Bax gene dosage. *PLoS Genet.* 1 17–26. 10.1371/journal.pgen.0010004 16103918 PMC1183523

[B156] Lin H.-J. Chen W.-C. Tsai F.-J. Tsai S.-W. (2002). Distributions of p53 codon 72 polymorphism in primary open angle glaucoma. *Br. J. Ophthalmol.* 86 767–770. 10.1136/bjo.86.7.767 12084746 PMC1771205

[B157] Lin W. Kuang H. (2014). Oxidative stress induces autophagy in response to multiple noxious stimuli in retinal ganglion cells. *Autophagy* 10 1692–1701. 10.4161/auto.36076 25207555 PMC4198355

[B158] Lindsey J. D. Duong-Polk K. X. Hammond D. Leung C. K. Weinreb R. N. (2015). Protection of injured retinal ganglion cell dendrites and unfolded protein response resolution after long-term dietary resveratrol. *Neurobiol. Aging* 36 1969–1981. 10.1016/j.neurobiolaging.2014.12.021 25772060

[B159] Liu L. Sun Q. Wang R. Chen Z. Wu J. Xia F. (2016). Methane attenuates retinal ischemia/reperfusion injury via anti-oxidative and anti-apoptotic pathways. *Brain Res.* 1646 327–333. 10.1016/j.brainres.2016.05.037 27208496

[B160] Liu X. Clark A. F. Wordinger R. J. (2007). Expression of ciliary neurotrophic factor (CNTF) and its tripartite receptor complex by cells of the human optic nerve head. *Mol. Vis.* 13 758–763. 17563726 PMC2768760

[B161] Lomb D. J. Desouza L. A. Franklin J. L. Freeman R. S. (2009). Prolyl hydroxylase inhibitors depend on extracellular glucose and hypoxia-inducible factor (HIF)-2 to inhibit cell death caused by nerve growth factor (NGF) deprivation: evidence that HIF-2 has a role in NGF-promoted survival of sympathetic neurons. *Mol. Pharmacol.* 75 1198–1209. 10.1124/mol.108.053157 19204094 PMC2672811

[B162] Lv B. Wang R. Gao X. Dong X. Ji X. (2014). Effect of vascular endothelial growth factor on retinal ganglion cells of rats with chronic intraocular hypertension. *Int. J. Clin. Exp. Pathol.* 7 5717–5724. 25337213 PMC4203184

[B163] Mabuchi F. Sakurada Y. Kashiwagi K. Yamagata Z. Iijima H. Tsukahara S. (2009). Lack of association between p53 gene polymorphisms and primary open angle glaucoma in the Japanese population. *Mol. Vis.* 15 1045–1049. 19471604 PMC2684750

[B164] Maffei L. Berardi N. Domenici L. Parisi V. Pizzorusso T. (1992). Nerve growth factor (NGF) prevents the shift in ocular dominance distribution of visual cortical neurons in monocularly deprived rats. *J. Neurosci.* 12 4651–4662. 10.1523/JNEUROSCI.12-12-04651.1992 1334503 PMC6575769

[B165] Majsterek I. Malinowska K. Stanczyk M. Kowalski M. Blaszczyk J. Kurowska A. K. (2011). Evaluation of oxidative stress markers in pathogenesis of primary open-angle glaucoma. *Exp. Mol. Pathol.* 90 231–237. 10.1016/j.yexmp.2011.01.001 21241689

[B166] Malik J. M. I. Shevtsova Z. Bähr M. Kügler S. (2005). Long-term in vivo inhibition of CNS neurodegeneration by Bcl-XL gene transfer. *Mol. Ther.* 11 373–381. 10.1016/j.ymthe.2004.11.014 15727933

[B167] Malinowska K. Kowalski M. Szaflik J. Szaflik J. P. Majsterek I. (2016). The role of Cat -262C/T, GPX1 Pro198Leu and Sod1+35A/C gene polymorphisms in a development of primary open-angle glaucoma in a Polish population. *Pol. J. Pathol.* 67 404–410. 10.5114/pjp.2016.65875 28547970

[B168] Man P. Y. W. Turnbull D. M. Chinnery P. F. (2002). Leber hereditary optic neuropathy. *J. Med. Genet.* 39 162–169. 10.1136/jmg.39.3.162 11897814 PMC1735056

[B169] Martínez-Moreno C. G. Calderón-Vallejo D. Harvey S. Arámburo C. Quintanar J. L. (2018). Growth hormone (GH) and gonadotropin-releasing hormone (GnRH) in the central nervous system: a potential neurological combinatory therapy? *Int. J. Mol. Sci.* 19:E375. 10.3390/ijms19020375 29373545 PMC5855597

[B170] Mathews M. K. Guo Y. Langenberg P. Bernstein S. L. (2015). Ciliary neurotrophic factor (CNTF)-mediated ganglion cell survival in a rodent model of non-arteritic anterior ischaemic optic neuropathy (NAION). *Br. J. Ophthalmol.* 99 133–137. 10.1136/bjophthalmol-2014-305969 25336580 PMC4477946

[B171] Mayer M. P. Bukau B. (2005). Hsp70 chaperones: cellular functions and molecular mechanism. *Cell. Mol. Life Sci.* 62 670–684. 10.1007/s00018-004-4464-6 15770419 PMC2773841

[B172] Mazan-Mamczarz K. Galban S. de Silanes I. L. Martindale J. L. Atasoy U. Keene J. D. (2003). RNA-binding protein HuR enhances p53 translation in response to ultraviolet light irradiation. *Proc. Natl. Acad. Sci. U.S.A.* 100 8354–8359. 10.1073/pnas.1432104100 12821781 PMC166233

[B173] Milani P. Amadio M. Laforenza U. Dell’Orco M. Diamanti L. Sardone V. (2013). Posttranscriptional regulation of SOD1 gene expression under oxidative stress: potential role of ELAV proteins in sporadic ALS. *Neurobiol. Dis.* 60 51–60. 10.1016/j.nbd.2013.08.005 23969235

[B174] Miraucourt L. S. Tsui J. Gobert D. Desjardins J.-F. Schohl A. Sild M. (2016). Endocannabinoid signaling enhances visual responses through modulation of intracellular chloride levels in retinal ganglion cells. *eLife* 5:e15932. 10.7554/eLife.15932 27501334 PMC4987138

[B175] Mochizuki H. Murphy C. J. Brandt J. D. Kiuchi Y. Russell P. (2012). Altered stability of mRNAs associated with glaucoma progression in human trabecular meshwork cells following oxidative stress. *Invest. Ophthalmol. Vis. Sci.* 53 1734–1741. 10.1167/iovs.12-7938 22395891 PMC3342790

[B176] Molina-Holgado F. Pinteaux E. Heenan L. Moore J. D. Rothwell N. J. Gibson R. M. (2005). Neuroprotective effects of the synthetic cannabinoid HU-210 in primary cortical neurons are mediated by phosphatidylinositol 3-kinase/AKT signaling. *Mol. Cell. Neurosci.* 28 189–194. 10.1016/j.mcn.2004.09.004 15607953

[B177] Moon C. Park M. H. Kim S. Y. Moon C. Bae Y. C. Moon J. I. L. (2013). Differential cell death and Bcl-2 expression in the mouse retina after glutathione decrease by systemic D,L-buthionine sulphoximine administration. *Mol. Cells* 35 235–242. 10.1007/s10059-013-2276-y 23430084 PMC3887915

[B178] Munaut C. Lambert V. Noël A. Frankenne F. Deprez M. Foidart J. M. (2001). Presence of oestrogen receptor type beta in human retina. *Br. J. Ophthalmol.* 85 877–882. 10.1136/bjo.85.7.877 11423466 PMC1724050

[B179] Nabors L. B. Gillespie G. Y. Harkins L. King P. H. (2001). HuR, a RNA stability factor, is expressed in malignant brain tumors and binds to adenine- and uridine-rich elements within the 3’ untranslated regions of cytokine and angiogenic factor mRNAs. *Cancer Res.* 61 2154–2161. 11280780

[B180] Nakashima K. I. Iwao K. Inoue T. Haga A. Tsutsumi T. Mochita M. I. (2018). Stimulation of the adenosine A3 receptor, not the A1 or A2 receptors, promote neurite outgrowth of retinal ganglion cells. *Exp. Eye Res.* 170 160–168. 10.1016/j.exer.2018.02.019 29486164

[B181] Nakazawa T. Takahashi H. Shimura M. (2006). Estrogen has a neuroprotective effect on axotomized RGCs through ERK signal transduction pathway. *Brain Res.* 1093 141–149. 10.1016/j.brainres.2006.03.084 16696958

[B182] Nasti R. Rossi D. Amadio M. Pascale A. Unver M. Y. Hirsch A. K. H. (2017). Compounds interfering with embryonic lethal abnormal vision (ELAV) protein-RNA complexes: an avenue for discovering new drugs. *J. Med. Chem.* 60 8257–8267. 10.1021/acs.jmedchem.6b01871 28587461

[B183] Neamatzadeh H. Soleimanizad R. Zare-Shehneh M. Gharibi S. Shekari A. Rahimzadeh A. B. (2015). Association between p53 codon 72 (Arg72pro) polymorphism and primary open-angle glaucoma in Iranian patients. *Iran. Biomed. J.* 19 51–56. 10.6091/ibj.1379.2014 25605490 PMC4322233

[B184] Nebbioso M. Scarsella G. Tafani M. Pescosolido N. (2013). Mechanisms of ocular neuroprotection by antioxidant molecules in animal models. *J. Biol. Regul. Homeost. Agents* 27 197–205. 23489699

[B185] Newman-Casey P. A. Talwar N. Nan B. Musch D. C. Pasquale L. R. Stein J. D. (2014). The potential association between postmenopausal hormone use and primary open-angle glaucoma. *JAMA Ophthalmol.* 132 298–303. 10.1001/jamaophthalmol.2013.7618 24481323 PMC4106136

[B186] Nishi R. (1994). Neurotrophic factors: two are better than one. *Science* 265 1052–1053. 10.1126/science.8066443 8066443

[B187] Nowak A. Majsterek I. Przybyłowska-Sygut K. Pytel D. Szymanek K. Szaflik J. (2015). Analysis of the expression and polymorphism of APOE, HSP, BDNF, and GRIN2B genes associated with the neurodegeneration process in the pathogenesis of primary open angle glaucoma. *Biomed Res. Int.* 2015:258281. 10.1155/2015/258281 25893192 PMC4393917

[B188] Nowak A. Przybylowska-Sygut K. Szymanek K. Szaflik J. Szaflik J. P. Majsterek I. (2014). The relationship of TP53 and GRIN2B gene polymorphisms with risk of occurrence and progression of primary open-angle glaucoma in a Polish population. *Pol. J. Pathol.* 65 313–321. 10.5114/pjp.2014.48193 25693086

[B189] Nucci C. Gasperi V. Tartaglione R. Cerulli A. Terrinoni A. Bari M. (2007). Involvement of the endocannabinoid system in retinal damage after high intraocular pressure-induced ischemia in rats. *Invest. Ophthalmol. Vis. Sci.* 48 2997–3004. 10.1167/iovs.06-1355 17591864

[B190] Nuzzi R. Tridico F. (2017). Glaucoma: biological trabecular and neuroretinal pathology with perspectives of therapy innovation and preventive diagnosis. *Front. Neurosci.* 11:494. 10.3389/fnins.2017.00494 28928631 PMC5591842

[B191] Oddone F. Roberti G. Micera A. Busanello A. Bonini S. Quaranta L. (2017). Exploring serum levels of brain derived neurotrophic factor and nerve growth factor across glaucoma stages. *PLoS One* 12:e0168565. 10.1371/journal.pone.0168565 28068360 PMC5221757

[B192] Ojino K. Shimazawa M. Izawa H. Nakano Y. Tsuruma K. Hara H. (2015). Involvement of endoplasmic reticulum stress in optic nerve degeneration after chronic high intraocular pressure in DBA/2J mice. *J. Neurosci. Res.* 93 1675–1683. 10.1002/jnr.23630 26271210

[B193] Olmos G. Lladó J. (2014). Tumor necrosis factor alpha: a link between neuroinflammation and excitotoxicity. *Mediators Inflamm.* 2014:861231. 10.1155/2014/861231 24966471 PMC4055424

[B194] Ozaita A. Puighermanal E. Maldonado R. (2007). Regulation of PI3K/Akt/GSK-3 pathway by cannabinoids in the brain. *J. Neurochem.* 102 1105–1114. 10.1111/j.1471-4159.2007.04642.x 17484726

[B195] Ozdemir G. Tolun F. I. Gul M. Imrek S. (2009). Retinal oxidative stress induced by intraocular hypertension in rats may be ameliorated by brimonidine treatment and n-acetyl cysteine supplementation. *J. Glaucoma* 18 662–665. 10.1097/IJG.0b013e31819c46b1 20010244

[B196] Panchal S. S. Patidar R. K. Jha A. B. Allam A. A. Ajarem J. Butani S. B. (2017). Anti-inflammatory and antioxidative stress effects of oryzanol in glaucomatous rabbits. *J. Ophthalmol.* 2017 1–9. 10.1155/2017/1468716 28168044 PMC5266835

[B197] Park H. Y. L. Kim J. H. Park C. K. (2012). Activation of autophagy induces retinal ganglion cell death in a chronic hypertensive glaucoma model. *Cell Death Dis.* 3:e290. 10.1038/cddis.2012.26 22476098 PMC3358006

[B198] Pascale A. Govoni S. (2012). The complex world of post-transcriptional mechanisms: is their deregulation a common link for diseases? Focus on ELAV-like RNA-binding proteins. *Cell. Mol. Life Sci.* 69 501–517. 10.1007/s00018-011-0810-7 21909784 PMC11114966

[B199] Pate D. W. Järvinen K. Urtti A. Jarho P. Järvinen T. (1995). Ophthalmic arachidonylethanolamide decreases intraocular pressure in normotensive rabbits. *Curr. Eye Res.* 14 791–797. 10.3109/02713689508995801 8529418

[B200] Pease M. E. Zack D. J. Berlinicke C. Bloom K. Cone F. Wang Y. (2009). Effect of CNTF on retinal ganglion cell survival in experimental glaucoma. *Invest. Ophthalmol. Vis. Sci.* 50 2194–2200. 10.1167/iovs.08-3013 19060281 PMC2810634

[B201] Pedersen M. Jensen R. Pedersen D. S. Skjolding A. D. Hempel C. Maretty L. (2009). Metallothionein-I+II in neuroprotection. *Biofactors* 35 315–325. 10.1002/biof.44 19655389

[B202] Pietrucha-Dutczak M. Smedowski A. Liu X. Matuszek I. Varjosalo M. Lewin-Kowalik J. (2017). Candidate proteins from predegenerated nerve exert time-specific protection of retinal ganglion cells in glaucoma. *Sci. Rep.* 7:14540. 10.1038/s41598-017-14860-5 29109409 PMC5673995

[B203] Pirhan D. Yüksel N. Emre E. Cengiz A. Kürşat Yildiz D. (2016). Riluzole- and resveratrol-induced delay of retinal ganglion cell death in an experimental model of glaucoma. *Curr. Eye Res.* 41 59–69. 10.3109/02713683.2015.1004719 25658983

[B204] Piri N. Kwong J. M. K. Gu L. Caprioli J. (2016). Heat shock proteins in the retina: focus on HSP70 and alpha crystallins in ganglion cell survival. *Prog. Retin. Eye Res.* 52 22–46. 10.1016/j.preteyeres.2016.03.001 27017896 PMC4842330

[B205] Prokai-Tatrai K. Xin H. Nguyen V. Szarka S. Blazics B. Prokai L. (2013). 17 beta-estradiol eye drops protect the retinal ganglion cell layer and preserve visual function in an in vivo model of glaucoma. *Mol. Pharm.* 10 3253–3261. 10.1021/mp400313u 23841874 PMC3758120

[B206] Quaranta L. Bettelli S. Uva M. G. Semeraro F. Turano R. Gandolfo E. (2003). Effect of *Ginkgo biloba* extract on preexisting visual field damage in normal tension glaucoma. *Ophthalmology* 110 359–362. 10.1016/S0161-6420(02)01745-1 12578781

[B207] Ramirez A. I. de Hoz R. Salobrar-Garcia E. Salazar J. J. Rojas B. Ajoy D. (2017). The role of microglia in retinal neurodegeneration: Alzheimer’s disease, Parkinson, and glaucoma. *Front. Aging Neurosci.* 9:214 10.3389/fnagi.2017.00214 PMC5498525 28729832

[B208] Ravagnan L. Gurbuxani S. Susin S. A. Maisse C. Daugas E. Zamzami N. (2001). Heat-shock protein 70 antagonizes apoptosis-inducing factor. *Nat. Cell Biol.* 3 839–843. 10.1038/ncb0901-839 11533664

[B209] Rhee K. D. Nusinowitz S. Chao K. Yu F. Bok D. Yang X.-J. (2013). CNTF-mediated protection of photoreceptors requires initial activation of the cytokine receptor gp130 in Muller glial cells. *Proc. Natl. Acad. Sci. U.S.A.* 110 E4520–E4529. 10.1073/pnas.1303604110 24191003 PMC3839707

[B210] Roberti G. Mantelli F. Macchi I. Massaro-Giordano M. Centofanti M. (2014). Nerve growth factor modulation of retinal ganglion cell physiology. *J. Cell. Physiol.* 229 1130–1133. 10.1002/jcp.24573 24501088

[B211] Roh M. Zhang Y. Murakami Y. Thanos A. Lee S. C. Vavvas D. G. (2012). Etanercept, a widely used inhibitor of tumor necrosis factor-α (TNF- α), prevents retinal ganglion cell loss in a rat model of glaucoma. *PLoS One* 7:e40065. 10.1371/journal.pone.0040065 22802951 PMC3388998

[B212] Rokicki W. Zalejska-Fiolka J. Pojda-Wilczek D. Hampel A. Majewski W. Ogultekin S. (2017). Differences in serum oxidative status between glaucomatous and nonglaucomatous cataract patients. *BMC Ophthalmol.* 17:13. 10.1186/s12886-017-0409-3 28202006 PMC5311834

[B213] Rong X. Mo X. Ren T. Yang S. Yuan W. Dong J. (2011). Neuroprotective effect of erythropoietin-loaded composite microspheres on retinal ganglion cells in rats. *Eur. J. Pharm. Sci.* 43 334–342. 10.1016/j.ejps.2011.05.011 21621611

[B214] Rudzinski M. Wong T. P. Saragovi H. U. (2004). Changes in retinal expression of neurotrophins and neurotrophin receptors induced by ocular hypertension. *J. Neurobiol.* 58 341–354. 10.1002/neu.10293 14750147

[B215] Russo R. Cavaliere F. Watanabe C. Nucci C. Bagetta G. Corasaniti M. T. (2008). 17Beta-estradiol prevents retinal ganglion cell loss induced by acute rise of intraocular pressure in rat. *Prog. Brain Res.* 173 583–590. 10.1016/S0079-6123(08)01144-8 18929136

[B216] Ryul Ahn H. Kim K. A. Kang S. W. Lee J. Y. Kim T. J. Jung S. H. (2017). Persimmon leaves (*Diospyros kaki*) extract protects optic nerve crush-induced retinal degeneration. *Sci. Rep.* 7:46449. 10.1038/srep46449 28425487 PMC5397840

[B217] Saccà S. C. Gandolfi S. Bagnis A. Manni G. Damonte G. Traverso C. E. (2016). From DNA damage to functional changes of the trabecular meshwork in aging and glaucoma. *Ageing Res. Rev.* 29 26–41. 10.1016/j.arr.2016.05.012 27242026

[B218] Sacca S. C. La Maestra S. Micale R. T. Larghero P. Travaini G. Baluce B. (2011). Ability of dorzolamide hydrochloride and timolol maleate to target mitochondria in glaucoma therapy. *Arch. Ophthalmol.* 129 48–55. 10.1001/archophthalmol.2010.324 21220628

[B219] Saint-Geniez M. Maharaj A. S. R. Walshe T. E. Tucker B. A. Sekiyama E. Kurihara T. (2008). Endogenous VEGF is required for visual function: evidence for a survival role on Müller cells and photoreceptors. *PLoS One* 3:e3554. 10.1371/journal.pone.0003554 18978936 PMC2571983

[B220] Sanders E. J. Parker E. Harvey S. (2006). Retinal ganglion cell survival in development: mechanisms of retinal growth hormone action. *Exp. Eye Res.* 83 1205–1214. 10.1016/j.exer.2006.06.009 16893540

[B221] Sanders E. J. Parker E. Harvey S. (2009). Endogenous growth hormone in human retinal ganglion cells correlates with cell survival. *Mol. Vis.* 15 920–926. 19421410 PMC2676198

[B222] Schultz R. Witte O. W. Schmeer C. (2016). Increased frataxin levels protect retinal ganglion cells after acute ischemia/reperfusion in the mouse retina in vivo. *Invest. Ophthalmol. Vis. Sci.* 57 4115–4124. 10.1167/iovs.16-19260 27537261

[B223] Schwitzer T. Schwan R. Angioi-Duprez K. Giersch A. Laprevote V. (2016). The endocannabinoid system in the retina: from physiology to practical and therapeutic applications. *Neural Plast* 2016:2916732. 10.1155/2016/2916732 26881099 PMC4736597

[B224] Sena D. F. Lindsley K. (2017). Neuroprotection for treatment of glaucoma in adults. *Cochrane Database Syst. Rev.* 2:CD006539. 10.1002/14651858.CD006539.pub4 23450569 PMC4261923

[B225] Shah S. S. Tsang S. H. Mahajan V. B. (2009). Erythropoietin receptor expression in the human diabetic retina. *BMC Res. Notes* 2:234. 10.1186/1756-0500-2-234 19930719 PMC2785834

[B226] Shaw P. X. Sang A. Wang Y. Ho D. Douglas C. Dia L. (2017). Topical administration of a Rock/Net inhibitor promotes retinal ganglion cell survival and axon regeneration after optic nerve injury. *Exp. Eye Res.* 158 33–42. 10.1016/j.exer.2016.07.006 27443501 PMC5821900

[B227] Sheth S. Brito R. Mukherjea D. Rybak L. P. Ramkumar V. (2014). Adenosine receptors: expression, function and regulation. *Int. J. Mol. Sci.* 15 2024–2052. 10.3390/ijms15022024 24477263 PMC3958836

[B228] Shih H. M. Wu C. J. Lin S. L. (2018). Physiology and pathophysiology of renal erythropoietin-producing cells. *J. Formos. Med. Assoc.* 117 955–963. 10.1016/j.jfma.2018.03.017 29655605

[B229] Shirley Ding S. L. Leow S. N. Munisvaradass R. Koh E. H. Bastion M. L. C. Then K. Y. (2016). Revisiting the role of erythropoietin for treatment of ocular disorders. *Eye* 30 1293–1309. 10.1038/eye.2016.94 27285322 PMC5129866

[B230] Shpak A. A. Guekht A. B. Druzhkova T. A. Kozlova K. I. Gulyaeva N. V. (2017). Ciliary neurotrophic factor in patients with primary open-angle glaucoma and age-related cataract. *Mol. Vis.* 23 799–809. 10.1080/02713683.2017.1396617 29225456 PMC5710971

[B231] Shruthi S. Sumitha R. Varghese A. M. Ashok S. Chandrasekhar Sagar B. K. Sathyaprabha T. N. (2016). Brain-derived neurotrophic factor facilitates functional recovery from ALS-cerebral spinal fluid-induced neurodegenerative changes in the NSC-34 motor neuron cell line. *Neurodegener. Dis.* 17 44–58. 10.1159/000447559 27617773

[B232] Skaper S. (2008). The biology of neurotrophins, signalling pathways, and functional peptide mimetics of neurotrophins and their receptors. *CNS Neurol. Disord. Drug Targets* 7 46–62. 10.2174/187152708783885174 18289031

[B233] Skliris A. Papadaki O. Kafasla P. Karakasiliotis I. Hazapis O. Reczko M. (2015). Neuroprotection requires the functions of the RNA-binding protein HuR. *Cell Death Differ.* 22 703–718. 10.1038/cdd.2014.158 25301069 PMC4392069

[B234] Smedowski A. Liu X. Podracka L. Akhtar S. Trzeciecka A. Pietrucha-Dutczak M. (2018). Increased intraocular pressure alters the cellular distribution of HuR protein in retinal ganglion cells – A possible sign of endogenous neuroprotection failure. *Biochim. Biophys. Acta Mol. Basis Dis.* 1864 296–306. 10.1016/j.bbadis.2017.10.030 29107807

[B235] Song W. Huang P. Zhang C. (2015). Neuroprotective therapies for glaucoma. *Drug Des. Devel. Ther.* 9 1469–1479. 10.2147/DDDT.S80594 25792807 PMC4362661

[B236] Soto I. Howell G. R. (2014). The complex role of neuroinflammation in glaucoma. *Cold Spring Harb. Perspect. Med.* 4:a017269. 10.1101/cshperspect.a017269 24993677 PMC4109578

[B237] Srivastava P. (2002). Roles of heat-shock proteins in innate and adaptive immunity. *Nat. Rev. Immunol.* 2 185–194. 10.1038/nri749 11913069

[B238] Su W. Li Z. Jia Y. Zhuo Y. (2014). Rapamycin is neuroprotective in a rat chronic hypertensive glaucoma model. *PLoS One* 9:e99719. 10.1371/journal.pone.0099719 24923557 PMC4055719

[B239] Suemori S. Shimazawa M. Kawase K. Satoh M. Nagase H. Yamamoto T. (2006). Metallothionein, an endogenous antioxidant, protects against retinal neuron damage in mice. *Invest. Ophthalmol. Vis. Sci.* 47 3975–3982. 10.1167/iovs.06-0275 16936113

[B240] Sugitani K. Koriyama Y. Sera M. Arai K. Ogai K. Wakasugi K. (2017). A novel function of neuroglobin for neuroregeneration in mice after optic nerve injury. *Biochem. Biophys. Res. Commun.* 493 1254–1259. 10.1016/j.bbrc.2017.09.127 28951213

[B241] Sutton A. Imbert A. Igoudjil A. Descatoire V. Cazanave S. Pessayre D. (2005). The manganese superoxide dismutase Ala16Val dimorphism modulates both mitochondrial import and mRNA stability. *Pharmacogenet. Genomics* 15 311–319. 10.1097/01213011-200505000-00006 15864132

[B242] Takayama S. Reed J. C. Homma S. (2003). Heat-shock proteins as regulators of apoptosis. *Oncogene* 22 9041–9047. 10.1038/sj.onc.1207114 14663482

[B243] Tan H. Kang X. Zhong Y. Shen X. Cheng Y. Jiao Q. (2012a). Erythropoietin upregulates growth associated protein-43 expression and promotes retinal ganglion cell axonal regeneration in vivo after optic nerve crush. *Neural Regen. Res.* 7 295–301. 10.3969/j.issn.1673-5374.2012.04.010 25806072 PMC4353103

[B244] Tan H. Zhong Y. Shen X. Cheng Y. Jiao Q. Deng L. (2012b). Erythropoietin promotes axonal regeneration after optic nerve crush in vivo by inhibition of RhoA/ROCK signaling pathway. *Neuropharmacology* 63 1182–1190. 10.1016/j.neuropharm.2012.06.037 22750411

[B245] Tao W. (2006). Application of encapsulated cell technology for retinal degenerative diseases. *Expert Opin. Biol. Ther.* 6 717–726. 10.1517/14712598.6.7.717 16805711

[B246] Tezel G. (2008). TNF-α signaling in glaucomatous neurodegeneration. *Prog. Brain Res.* 173 409–421. 10.1016/S0079-6123(08)01128-X 18929124 PMC3150483

[B247] Tezel G. Wax M. B. (2000). Increased production of tumor necrosis factor-alpha by glial cells exposed to simulated ischemia or elevated hydrostatic pressure induces apoptosis in cocultured retinal ganglion cells. *J. Neurosci.* 20 8693–8700. 10.1523/JNEUROSCI.20-23-08693.2000 11102475 PMC6773089

[B248] Thanos C. G. Bell W. J. O’Rourke P. Kauper K. Sherman S. Stabila P. (2004). Sustained secretion of ciliary neurotrophic factor to the vitreous, using the encapsulated cell therapy-based NT-501 intraocular device. *Tissue Eng.* 10 1617–1622. 10.1089/ten.2004.10.1617 15684670

[B249] Tian C. Fang S. Du X. Jia C. (2011). Association of the C47T polymorphism in SOD2 with diabetes mellitus and diabetic microvascular complications: a meta-analysis. *Diabetologia* 54 803–811. 10.1007/s00125-010-2004-5 21181397

[B250] Tieger M. G. Hedges T. R. Ho J. Erlich-Malona N. K. Vuong L. N. Athappilly G. K. (2017). Ganglion cell complex loss in chiasmal compression by brain tumors. *J. Neuroophthalmol.* 37 7–12. 10.1097/WNO.0000000000000424 28192385 PMC6033516

[B251] Vajaranant T. S. Pasquale L. R. (2013). Estrogen deficiency accelerates aging of the optic nerve. *Menopause* 19 942–947. 10.1097/gme.0b013e3182443137.Estrogen 22415565 PMC3376696

[B252] Vecino E. Ugarte M. Nash M. S. Osborne N. N. (1999). NMDA induces BDNF expression in the albino rat retina in vivo. *Neuroreport* 10 1103–1106. 10.1097/00001756-199904060-00036 10321491

[B253] Viiri J. Amadio M. Marchesi N. Hyttinen J. M. T. Kivinen N. Sironen R. (2013). Autophagy activation clears ELAVL1/HuR-mediated accumulation of SQSTM1/p62 during proteasomal inhibition in human retinal pigment epithelial cells. *PLoS One* 8:e69563. 10.1371/journal.pone.0069563 23922739 PMC3726683

[B254] Wan P. Su W. Zhang Y. Li Z. Deng C. Zhuo Y. (2017). Trimetazidine protects retinal ganglion cells from acute glaucoma via the Nrf2/Ho-1 pathway. *Clin. Sci.* 131 2363–2375. 10.1042/CS20171182 28811386 PMC5582167

[B255] Wang H. Wang R. Thrimawithana T. Little P. J. Xu J. Feng Z. P. (2014). The nerve growth factor signaling and its potential as therapeutic target for glaucoma. *Biomed Res. Int.* 2014:759473. 10.1155/2014/759473 25250333 PMC4164261

[B256] Wang Y. Zhang H. Liu Y. Li P. Cao Z. Cao Y. (2014). Erythropoietin (EPO) protects against high glucose-induced apoptosis in retinal ganglional cells. *Cell Biochem. Biophys.* 71 749–755. 10.1007/s12013-014-0259-z 25287674

[B257] Wang S. K. Chang R. T. (2014). An emerging treatment option for glaucoma: Rho kinase inhibitors. *Clin. Ophthalmol.* 8 883–890. 10.2147/OPTH.S41000 24872673 PMC4025933

[B258] Wartmann M. Campbell D. Subramanian A. Burstein S. H. Davis R. J. (1995). The MAP kinase signal transduction pathway is activated by the endogenous cannabinoid anandamide. *FEBS Lett.* 359 133–136. 10.1016/0014-5793(95)00027-7 7867785

[B259] Wei X. Yu Z. Cho K. S. Chen H. Malik M. T. A. Chen X. (2011). Neuroglobin is an endogenous neuroprotectant for retinal ganglion cells against glaucomatous damage. *Am. J. Pathol.* 179 2788–2797. 10.1016/j.ajpath.2011.08.015 21967817 PMC3260831

[B260] Wei Y. Gong J. Yoshida T. Eberhart C. G. Xu Z. Kombairaju P. (2011). Nrf2 has a protective role against neuronal and capillary degeneration in retinal ischemia-reperfusion injury. *Free Radic. Biol. Med.* 51 216–224. 10.1016/j.freeradbiomed.2011.04.026 21545836 PMC3997112

[B261] Weinreb R. N. Liebmann J. M. Cioffi G. A. Goldberg I. Brandt J. D. Johnson C. A. (2018). Oral memantine for the treatment of glaucoma: design and results of 2 randomized, placebo-controlled, phase 3 studies. *Ophthalmology* 10.1016/j.ophtha.2018.06.017 [Epub ahead of print]. 30082073

[B262] Weissmiller A. M. Wu C. (2012). Current advances in using neurotrophic factors to treat neurodegenerative disorders. *Transl. Neurodegener.* 1:14. 10.1186/2047-9158-1-14 23210531 PMC3542569

[B263] Wen R. Song Y. Liu Y. Li Y. Zhao L. Laties A. M. (2008). CNTF Negatively regulates the phototransduction machinery in rod photoreceptors: implication for light-induced photostasis plasticity. *Adv. Exp. Med. Biol.* 613 407–413. 10.1007/978-0-387-74904-4_48 18188971

[B264] Williams P. A. Marsh-Armstrong N. Howell G. R. Bosco A. Danias J. Simon J. (2017). Neuroinflammation in glaucoma: a new opportunity. *Exp. Eye Res.* 157 20–27. 10.1016/j.exer.2017.02.014 28242160 PMC5497582

[B265] Wilson G. N. Inman D. M. Denger-Crish C. M. Smith M. A. Crish S. D. (2015). Early pro-inflammatory cytokine elevations in the DBA/2J mouse model of glaucoma. *J. Neuroinflammation* 12:176. 10.1186/s12974-015-0399-0 26376776 PMC4574349

[B266] WoldeMussie E. Yoles E. Schwartz M. Ruiz G. Wheeler L. A. (2002). Neuroprotective effect of memantine in different retinal injury models in rats. *J. Glaucoma* 11 474–480. 10.1097/00061198-200212000-00003 12483089

[B267] Wolf C. Gramer E. Müller-Myhsok B. Pasutto F. Reinthal E. Wissinger B. (2009). Evaluation of nine candidate genes in patients with normal tension glaucoma: a case control study. *BMC Med. Genet.* 10:91. 10.1186/1471-2350-10-91 19754948 PMC2751751

[B268] Xin X. Gao L. Wu T. Sun F. (2013). Roles of tumor necrosis factor alpha gene polymorphisms, tumor necrosis factor alpha level in aqueous humor, and the risks of open angle glaucoma: a meta-analysis. *Mol. Vis.* 19 526–535. 23559847 PMC3611941

[B269] Xu C. Bailly-Maitre B. Reed J. C. (2005). Endoplasmic reticulum stress: cell life and death decisions. *J. Clin. Invest.* 115 2656–2664. 10.1172/JCI26373 16200199 PMC1236697

[B270] Xu J. Y. Chen C. (2015). Endocannabinoids in synaptic plasticity and neuroprotection. *Neuroscientist* 21 152–168. 10.1177/1073858414524632 24571856 PMC4143501

[B271] Xu Z. Cho H. Hartsock M. J. Mitchell K. L. Gong J. Wu L. (2015). Neuroprotective role of Nrf2 for retinal ganglion cells in ischemia-reperfusion. *J. Neurochem.* 133 233–241. 10.1111/jnc.13064 25683606 PMC4413918

[B272] Yan Q. Wang J. Matheson C. R. Urich J. L. (1999). Glial cell line-derived neurotrophic factor (GDNF) promotes the survival of axotomized retinal ganglion cells in adult rats: comparison to and combination with brain-derived neurotrophic factor (BDNF). *J. Neurobiol.* 38 382–390. 10.1002/(SICI)1097-4695(19990215)38:3<382::AID-NEU7>3.0.CO;2-5 10022580

[B273] Yang L. Li S. Miao L. Huang H. Liang F. Teng X. (2016). Rescue of glaucomatous neurodegeneration by differentially modulating neuronal endoplasmic reticulum stress molecules. *J. Neurosci.* 36 5891–5903. 10.1523/JNEUROSCI.3709-15.2016 27225776 PMC4879204

[B274] Yang X. Luo C. Cai J. Powell D. W. Yu D. Kuehn M. H. (2011). Neurodegenerative and inflammatory pathway components linked to TNF-α/TNFR1 signaling in the glaucomatous human retina. *Invest. Ophthalmol. Vis. Sci.* 52 8442–8454. 10.1167/iovs.11-8152 21917936 PMC3208177

[B275] Yazihan N. Karakurt O. Ataoglu H. (2008). Erythropoietin reduces lipopolysaccharide-induced cell Damage and midkine secretion in U937 human histiocytic lymphoma cells. *Adv. Ther.* 25 502–514. 10.1007/s12325-008-0055-5 18465096

[B276] Yildirim Z. Kilic N. Ozer C. Babul A. Take G. Erdogan D. (2007). Effects of taurine in cellular responses to oxidative stress in young and middle-aged rat liver. *Ann. N. Y. Acad. Sci.* 1100 553–561. 10.1196/annals.1395.061 17460221

[B277] Yu Z. Liu N. Wang X. (2012). “Neuroglobin: a novel target for endogenous neuroprotection,” in *Translational Stroke Research* ed. Zhang J. H. (New York, NY: Springer) 353–372. 10.1007/978-1-4419-9530-8_18

[B278] Yu-Wai-Man P. Soiferman D. Moore D. G. Burté F. Saada A. (2017). Evaluating the therapeutic potential of idebenone and related quinone analogues in Leber hereditary optic neuropathy. *Mitochondrion* 36 36–42. 10.1016/j.mito.2017.01.004 28093355 PMC5644719

[B279] Zachary I. (2005). Neuroprotective role of vascular endothelial growth factor: signalling mechanisms, biological function, and therapeutic potential. *Neurosignals* 14 207–221. 10.1159/000088637 16301836

[B280] Zhang M. Hu H. Zhang X. Lu W. Lim J. Eysteinsson T. (2010). The A3 adenosine receptor attenuates the calcium rise triggered by NMDA receptors in retinal ganglion cells. *Neurochem. Int.* 56 35–41. 10.1016/j.neuint.2009.08.011 19723551 PMC2814940

[B281] Zhang X. Zhang M. Laties A. M. Mitchell C. H. (2006). Balance of purines may determine life or death of retinal ganglion cells as A3 adenosine receptors prevent loss following P2X7 receptor stimulation. *J. Neurochem.* 98 566–575. 10.1111/j.1471-4159.2006.03900.x 16805847

[B282] Zhang Y. Chen P. Di G. Qi X. Zhou Q. Gao H. (2018). Netrin-1 promotes diabetic corneal wound healing through molecular mechanisms mediated via the adenosine 2B receptor. *Sci. Rep.* 8:5994. 10.1038/s41598-018-24506-9 29662125 PMC5902612

[B283] Zhong L. Bradley J. Schubert W. Ahmed E. Adamis A. P. Shima D. T. (2007). Erythropoietin promotes survival of retinal ganglion cells in DBA/2J glaucoma mice. *Invest. Ophthalmol. Vis. Sci.* 48 1212–1218. 10.1167/iovs.06-0757 17325165

[B284] Zhong Y. Yang Z. Huang W. C. Luo X. (2013). Adenosine, adenosine receptors and glaucoma: an updated overview. *Biochim. Biophys. Acta* 1830 2882–2890. 10.1016/j.bbagen.2013.01.005 23328492

[B285] Zhong Y.-S. Liu X.-H. Cheng Y. Min Y.-J. (2008). Erythropoietin with retrobulbar administration protects retinal ganglion cells from acute elevated intraocular pressure in rats. *J. Ocul. Pharmacol. Ther.* 24 453–459. 10.1089/jop.2008.0021 18788995

[B286] Zhou X. Li F. Ge J. Sarkisian S. R. Tomita H. Zaharia A. (2007). Retinal ganglion cell protection by 17-beta-estradiol in a mouse model of inherited glaucoma. *Dev. Neurobiol.* 67 603–616. 10.1002/dneu.20373 17443811

[B287] Zucal C. D’Agostino V. Loffredo R. Mantelli B. Thongon N. Lal P. (2015). Targeting the multifaceted HuR protein, benefits and caveats. *Curr. Drug Targets* 16 499–515. 10.2174/1389450116666150223163632 25706256

